# Supplementary material for: Prevalence and access to care for cardiovascular risk factors in older people in Sierra Leone: a cross-sectional survey
Source: BMJ Open. 2020 Sep 9;10(9):e038520. doi: 10.1136/bmjopen-2020-038520 (PMC7482482; doi:10.1136/bmjopen-2020-038520)
Supplement: Supplementary data [file bmjopen-2020-038520supp001.pdf]

Appendix figure 1 Map of Bo Districts, Sierra Leone

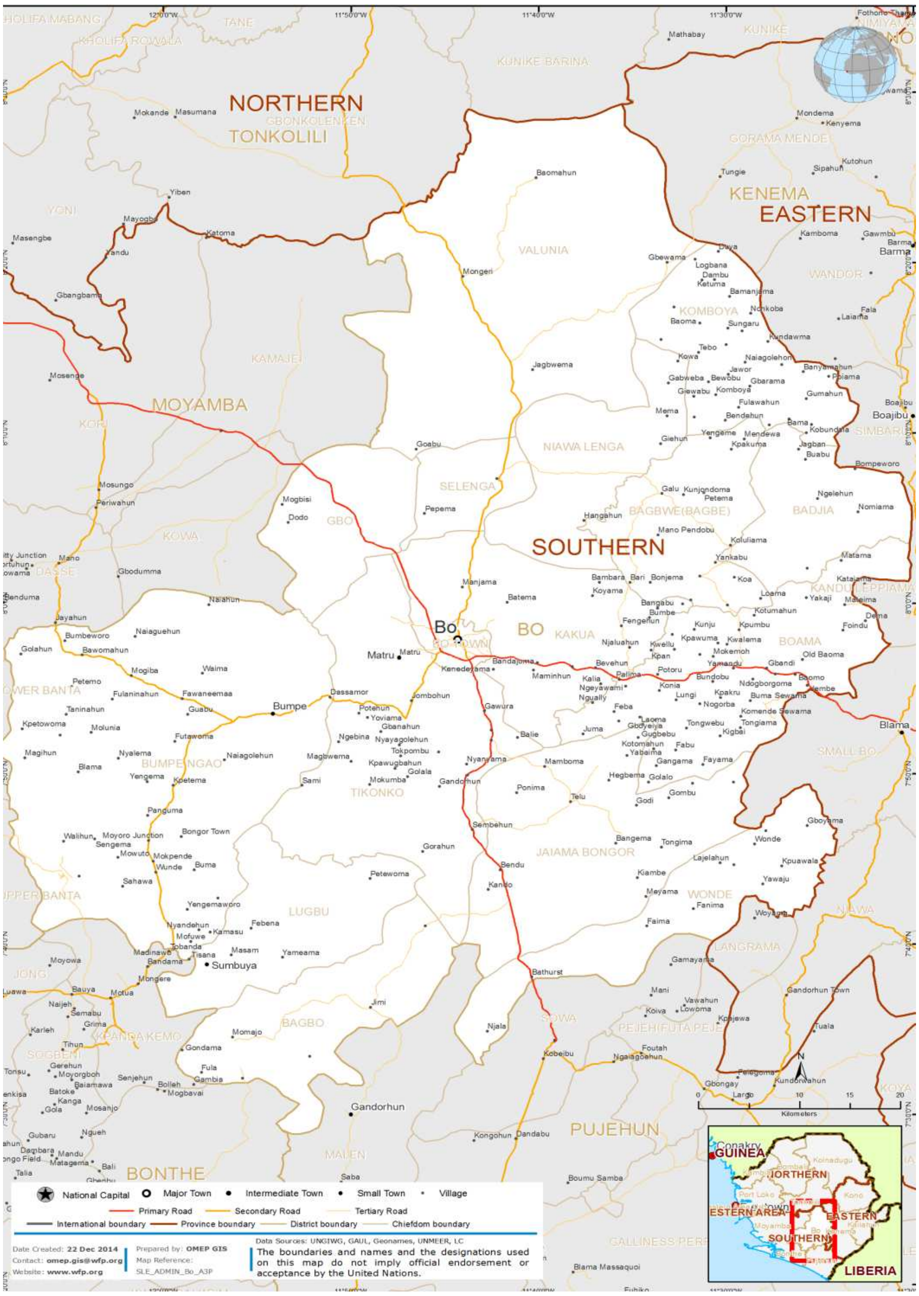

Appendix table 1 Sensitivity analysis with BMI >30 of multivariable associations between demographic characteristics and cardiovascular risk (n=2071)

parameter	group	Hypertension		Diabetes		Dyslipidaemia		Obesity (BMI>30)		Smoking		Total CVD risk factors incl. chol		Total CVD risk factors exl. chol	
		OR (95% CI)	P-value	OR (95% CI)	P-value	OR (95% CI)	P-value	OR (95% CI)	P-value	OR (95% CI)	P-value	OR (95% CI)	P-value	OR (95% CI)	P-value
Place of living	Rural	Referent	-	Referent	-	Referent	-	Referent	-	Referent	-	Referent	-	Referent	-
	Urban	1.04 (1.01-1.08)	0.014	1.46 (1.34-1.60)	<0.001	0.84 (0.75-0.93)	0.001	1.4 (1.33-1.48)	<0.001	1.13 (1.08-1.17)	<0.001	0.99 (0.93-1.05)	0.614	1.06 (1.02-1.10)	0.002
Gender	Female	Referent	-	Referent	-	Referent	-	Referent	-	Referent	-	Referent	-	Referent	-
	Male	0.78 (0.75-0.80)	<0.001	0.75 (0.69-0.82)	<0.001	0.88 (0.80-0.97)	0.013	0.24 (0.23-0.26)	<0.001	9.15 (8.76-9.54)	<0.001	1.6 (1.52-1.70)	<0.001	1.43 (1.38-1.48)	<0.001
Age	40-49	Referent	-	Referent	-	Referent	-	Referent	-	Referent	-	Referent	-	Referent	-
	50-59	1.75 (1.69-1.81)	<0.001	2.10 (1.91-2.32)	<0.001	1.38 (1.25-1.53)	<0.001	0.74 (0.70-0.79)	<0.001	0.84 (0.81-0.880	<0.001	0.93 (0.88-0.99)	0.023	1.15 (1.11-1.20)	<0.001
	60-69	2.35 (2.26-2.45)	<0.001	2.77 (2.50-3.07)	<0.001	1.36 (1.22-1.53)	<0.001	0.85 (0.80-0.91)	<0.001	0.58 (0.56-0.61)	<0.001	1.25 (1.16-1.35)	<0.001	1.70 (1.60-1.81)	<0.001
	70-79	3.43 (3.27-3.61)	<0.001	3.46 (3.07-3.89)	<0.001	1.76 (1.52-2.05)	<0.001	0.64 (0.59-0.70)	<0.001	0.36 (0.33-0.38)	<0.001	2.24 (2.00-2.51)	<0.001	1.25 (1.16-1.34)	<0.001
	>80	3.13 (2.96-3.32)	<0.001	1.76 (1.69-1.99)	<0.001	0.98 (0.81-1.19)	0.835	0.59 (0.53-0.66)	<0.001	0.52 (0.48-0.56)	<0.001	1.22 (1.09-1.38)	0.001	1.07 (1.16-1.34)	<0.001
Education level	No complete education	Referent	-	Referent	-	Referent	-	Referent	-	Referent		Referent		Referent	-
	Any education	1.17 (1.14-1.21)	<0.001	1.83 (1.69-1.99)	<0.001	1.08 (0.98-1.18)	0.111	1.26 (1.20-1.33)	<0.001	0.86 (0.83-0.89)	<0.001	0.91 (0.86-0.96)	0.001	1.07 (1.04-1.11)	<0.001
Marital status	Single/divorced/widow	Referent	-	Referent	-	Referent	-	Referent	-	Referent	-	Referent	-	Referent	-
	Married/Cohabiting	0.8 (0.78-0.83)	<0.001	1.01 (0.93-1.11)	0.785	0.62 (0.56-0.68)	<0.001	1.26 (1.20-1.33)	<0.001	0.8 (0.77-0.84)	<0.001	0.81 (0.76-0.86)	<0.001	0.84 (0.81-0.88)	<0.001

Wealth quintile	1	Referent	-	Referent	-	Referent	-	Referent	-	Referent	-	Referent	-	Referent	-
	2	0.83 (0.79-0.86)	<0.001	1.31 (1.14-1.52)	<0.001	1.84 (1.47-2.29)	<0.001	2 (1.83-2.20)	<0.001	0.96 (0.92-1.01)	0.105	1.06 (0.98-1.14)	0.142	1.1 (1.05-1.15)	<0.001
	3	0.99 (0.95-1.03)	0.698	1.2 (1.04-1.39)	0.014	2.36 (1.90-2.29)	<0.001	2.08 (1.90-2.78)	<0.001	0.71 (0.68-0.75)	<0.001	0.87 (0.81-0.94)	0.001	0.91 (0.87-0.95)	0.001
	4	1.3 (1.25-1.36)	<0.001	1.64 (1.42-1.88)	<0.001	6.19 (5.07-7.56)	<0.001	3.26 (2.98-3.55)	<0.001	0.51 (0.49-0.54)	<0.001	1.41 (1.30-1.53)	<0.001	1.32 (1.25-1.38)	<0.001
	5	1.6 (1.52-1.69)	<0.001	2.7 (2.34-3.12)	<0.001	11.16 (9.05-13.76)	<0.001	4.5 (4.12-4.93)	<0.001	0.39 (0.37-0.42)	<0.001	2.46 (2.23-2.73)	<0.001	1.62 (1.53-1.72)	<0.001

Appendix table 2 Demographic characteristics of participants with and without measured cholesterol (n = 2071)

parameter	group	Not measured cholesterol	Measured Cholesterol
Place of living	Rural	61.30%	61.50%
	Urban	38.70%	38.50%
Gender	Male	47.30%	40.60%
	Female	52.70%	59.4%*
Age median (IQR)		56 (46-67)	55 (47-68)
Education level	No completed education	70.10%	67.50%
	Any education	29.90%	32.30%
Marital status	Married/Cohabiting	69.20%	67.90%
	Single/widowed/divorced	30.80%	32.10%
Wealth quintile	1	21.10%	18.20%
	2	20.10%	19.80%
	3	20.80%	19.00%
	4	17.90%	23.10%
	5	20.10%	19.80%

*p<0.005

Appendix table 3 Univariable associations between demographic characteristics and cardiovascular risk factors (n=2071)

parameter	group	Hypertension	Diabetes	Hypercholesterolaemia	Obesity	Smoking	One CVD risk factor or incl. Cholesterol	One CVD risk factors or more exc. Cholesterol
Place of living	Rural	46.00%	2.30%	5.00%	20.40%	28.40%	75.50%	72.60%
	Urban	55.8%*	5.5%*	9.6%*	37.2%*	21.0%*	79.6%*	77.8%*
Gender	Female	54.80%	4.00%	7.90%	36.70%	8.40%	74.80%	72.30%
	Male	44.7%*	3.0%*	5.3%*	17.0%*	42.2%*	79.5%*	76.7%*
Age		0.239*	0.081*	0.080*	-0.039*	-0.114*	0.068*	0.073*
Age groups	40-49	37.50%	1.80%	4.90%	28.00%	30.60%	75.00%	71.60%
	50-59	52.20%	4.20%	7.30%	27.20%	27.60%	75.40%	75.70%
	60-69	60.50%	5.70%	9.80%	28.20%	19.10%	80.60%	76.30%
	70-79	68.00%	5.70%	9.90%	21.70%	14.70%	84.40%	80.40%
	>80	68.0%**	3.4%**	5.8%**	16.0%**	17.6%**	80.6%**	77.2%**
Education level	No completed education	48.50%	2.60%	5.60%	22.80%	24.60%	76.00%	72.90%
	Any education	52.0%*	5.4%*	8.9%*	34.3%*	27.8%*	79.1%*	77.7%*
Marital status	Married/Cohabiting	45.50%	3.20%	5.70%	25.50%	29.20%	76.30%	73.70%
	Single/divorced/ Widowed	60.6%*	4.3%*	9.4%*	29.4%*	16.1%*	79.1%*	76.7%*
Wealth quintile	1	45.80%	1.70%	1.60%	13.80%	32.00%	73.40%	71.10%
	2	41.80%	2.30%	3.10%	17.50%	32.10%	75.70%	73.40%
	3	46.90%	2.40%	3.80%	21.50%	26.60%	71.50%	70.20%
	4	54.00%	3.70%	9.50%	34.60%	20.40%	79.10%	77.50%
	5	62.4%**	7.7%**	15.0%**	50.6%**	16.0%**	86.3%**	82.2%**

*P<0.001, **P for trend<0.001
