# Supplementary material for: Prevalence and access to care for cardiovascular risk factors in older people in Sierra Leone: a cross-sectional survey
Source: BMJ Open. 2020 Sep 9;10(9):e038520. doi: 10.1136/bmjopen-2020-038520 (PMC7482482; doi:10.1136/bmjopen-2020-038520)
Supplement: Author's manuscript [file bmjopen-2020-038520.draft_revisions.pdf]

BMJ Open is committed to open peer review. As part of this commitment we make the peer review history of every article we publish publicly available.

When an article is published we post the peer reviewers' comments and the authors' responses online. We also post the versions of the paper that were used during peer review. These are the versions that the peer review comments apply to.

The versions of the paper that follow are the versions that were submitted during the peer review process. They are not the versions of record or the final published versions. They should not be cited or distributed as the published version of this manuscript.

BMJ Open is an open access journal and the full, final, typeset and author-corrected version of record of the manuscript is available on our site with no access controls, subscription charges or pay-per-view fees (http://bmjopen.bmj.com).

If you have any questions on BMJ Open's open peer review process please email info.bmjopen@bmj.com

BMJ Open

Prevalence and access to care for cardiovascular risk factors in older people in Sierra Leone: A cross-sectional survey

Journal:	*BMJ Open*
Manuscript ID	bmjopen-2020-038520
Article Type:	Original research
Date Submitted by the Author:	16-Mar-2020
Complete List of Authors:	Odland, Maria Lisa; University of Birmingham Institute of Applied Health Research, Department of Public Health and Nursing<br>Bockarie, Tahir; University of Warwick Warwick Medical School<br>Wurie, Haja; University of Sierra Leone College of Medicine and Allied Health Sciences<br>Ansumana, Rashid; Mercy Hospital Research Laboratory<br>Lamin, Joseph; Mercy Hospital Research Laboratory<br>Nugent, Rachel; RTI International<br>Bakolis, Ioannis; King's College London, London, United Kingdom, Centre for Implementation Science, Health Services and Population Research Department, Institute of Psychiatry, Psychology and Neuroscience, ; Department of Biostatistics and Health Informatics, Institute of Psychiatry, Psychology and Neuroscience, King's College London, London, United Kingdom<br>Witham, Miles; AGE Research Group, NIHR Newcastle Biomedical Research Centre, Newcastle University; Newcastle Upon Tyne Hospitals NHS Foundation Trust<br>Davies, Justine ; University of Birmingham Institute of Applied Health Research; King's College London, Centre for Global Health
Keywords:	Health policy < HEALTH SERVICES ADMINISTRATION & MANAGEMENT, Quality in health care < HEALTH SERVICES ADMINISTRATION & MANAGEMENT, Organisation of health services < HEALTH SERVICES ADMINISTRATION & MANAGEMENT, EPIDEMIOLOGY, PUBLIC HEALTH

SCHOLARONE™
Manuscripts

I, the Submitting Author has the right to grant and does grant on behalf of all authors of the Work (as defined in the below author licence), an exclusive licence and/or a non-exclusive licence for contributions from authors who are: i) UK Crown employees; ii) where BMJ has agreed a CC-BY licence shall apply, and/or iii) in accordance with the terms applicable for US Federal Government officers or employees acting as part of their official duties; on a worldwide, perpetual, irrevocable, royalty-free basis to BMJ Publishing Group Ltd ("BMJ") its licensees and where the relevant Journal is co-owned by BMJ to the co-owners of the Journal, to publish the Work in this journal and any other BMJ products and to exploit all rights, as set out in our licence.

The Submitting Author accepts and understands that any supply made under these terms is made by BMJ to the Submitting Author unless you are acting as an employee on behalf of your employer or a postgraduate student of an affiliated institution which is paying any applicable article publishing charge ("APC") for Open Access articles. Where the Submitting Author wishes to make the Work available on an Open Access basis (and intends to pay the relevant APC), the terms of reuse of such Open Access shall be governed by a Creative Commons licence – details of these licences and which Creative Commons licence will apply to this Work are set out in our licence referred to above.

Other than as permitted in any relevant BMJ Author's Self Archiving Policies, I confirm this Work has not been accepted for publication elsewhere, is not being considered for publication elsewhere and does not duplicate material already published. I confirm all authors consent to publication of this Work and authorise the granting of this licence.

For peer review only

Prevalence and access to care for cardiovascular risk factors in older people in Sierra Leone: A cross-sectional survey

Maria Lisa Odland,[1] Tahir Bockarie,[2] Haja Wurie,[3] Rashid Ansumana,[4] Joseph Lamin,[4] Rachel Nugent,[5] Ioannis Bakolis,[6,7] Miles Witham*[8,9] and Justine Davies*[9,10]

*Joint last co-authorship

[1] University of Birmingham Institute of Applied Health Research, Department of Public Health and Nursing Birmingham, West Midlands, UK

[2] University of Warwick, Warwick Medical School, Coventry, UK

[3] University of Sierra Leone College of Medicine and Allied Health Sciences, Freetown, Western Area, SL

[4] Mercy Hospital Research Laboratory, Freetown, Sierra Leone

[5] RTI International, Seattle, WA, USA

[6] King's College London, London, United Kingdom, Centre for Implementation Science, Health Services and Population Research Department, Institute of Psychiatry, Psychology and Neuroscience, London, London, UK

[7] Department of Biostatistics and Health Informatics, Institute of Psychiatry, Psychology and Neuroscience, King's College London, London, United Kingdom London, UK

[8] AGE Research Group, NIHR Newcastle Biomedical Research Centre, Newcastle University Newcastle upon Tyne, UK

[9] Newcastle Upon Tyne Hospitals NHS Foundation Trust Newcastle Upon Tyne, Newcastle upon Tyne , UK

[10] University of Birmingham Institute of Applied Health Research, Birmingham, West Midlands, UK

[11] King's College London, Centre for Global Health, London, UK

Corresponding author:

Maria Lisa Odland, Institute of Applied Health Research, College of Medical and Dental Sciences, University of Birmingham, Birmingham, B15 2TT, United Kingdom. Email: M.L.Odland@bham.ac.uk.

Word count: 4635 excluding strengths, abstract and references.

ABSTRACT

Introduction Prevalence of cardiovascular disease risk factors (CVDRF) is increasing, especially in low-income countries. In Sierra Leone, there is limited empirical data on the prevalence of CVDRFs, and there are no previous studies on the access to care for these.

Methods This study in rural and urban Sierra Leone collected demographic, anthropometric measurements, and clinical data from randomly sampled individuals over 40 years old using a household survey. We describe prevalence of CVDRFs diabetes, hypertension, dyslipidaemia, overweight or obesity, and smoking, or having at least one of these risk factors. Cascades of care were constructed for diabetes and hypertension, using % of the population with the disease who self-reported to have been screened, diagnosed and treated, or were controlled to target. Prevalence of CVDRFs and progress through the cascade for hypertension were associated with demographic and socio-economic variables using multivariable regression. Reasons for not accessing care were explored.

Results Of 2071 people, nearly 50% (49.6%, 95% CI 44.1-44.7) of the population had hypertension, 3.5% (3.4-3.6) had diabetes, 6.7% (6.5-7.0) had dyslipidaemia, 25.6% (25.4-25.9) smoked, and 26.5% (26.3-26.8%) were overweight/obese; 77.1% (76.6-77.5%) had at least one CVDRF. Regression models showed people in urban areas were more likely to have diabetes and be overweight. Moreover, being female, more educated, or wealthier increased the risk of having all CVDRFs except for smoking. There is substantial loss of patients at

For peer review only

each step of the care cascade for both diabetes and hypertension with less than 10% of the total population with the conditions being screened, diagnosed, treated and controlled. The most common reasons for not seeking care were lack of knowledge and cost.

Conclusions This is the first study to show that in Sierra Leone, CVDRFs are prevalent and access to care is low. Health system strengthening with a focus on CVDRFs is urgently needed.

For peer review only

Strengths and limitations

- This study is one of the first studies to report prevalence of multiple cardiovascular risk factors (CVDRFs) in such a large sample from Sierra Leone.
- This is the first study to report access to care for CVDRFs in Sierra Leone.
- The study sample is larger than any previous studies on CVDRF in Sierra Leone, and the data sampling and analysis were done in a rigorous way to avoid potential biases.
- The data collection was limited to a geographical area due to accessibility and travel times.

For peer review only

INTRODUCTION

Non-communicable diseases (NCDs) such as cardiovascular disease and its risk factors are major health problems globally.[1] The reduction in deaths from infections including HIV, together with lifestyle transitions towards a high-calorie, low-activity, urban lifestyle, have already led to a high and rising prevalence of NCDs in lower and middle income countries (LMIC).[2-5] In fact, high blood pressure has become the largest contributor to premature mortality globally,[2 3] and cardiovascular diseases (including coronary heart disease and stroke) are the most common NCDs, globally responsible for an estimated 17.8 million deaths in 2017.[6] More than three quarters of these were in LMICs. [6]

However, surveillance of the prevalence of cardiovascular disease risk factors is very limited in the poorest countries in the world. Sierra Leone is a low-income country situated in West-Africa. It has a human development index of 0.419 (184 of 189 countries)[7 8] and a maternal mortality ratio (1360 per 100 000 live births) and under-5 mortality rate (110.5 per 1000 live births) among the highest in the world.[9 10] The civil war from 1991-2002 disrupted infrastructure development, including that of the health system. Moreover, the 2013-2016 Ebola virus disease created a public health crisis and drew resources away from broader development of the health system.[11 12]

In recent years, both gross domestic product (GDP) and life expectancy at birth have increased in Sierra Leone.[7] In other countries (including those in sub-Saharan Africa) that have undergone a demographic transition, it has been accompanied by an increasing burden of cardiovascular disease risk factors (CVDRF) – such as diabetes and hypertension, dyslipidaemia, obesity and overweight – with consequent macro- and microvascular disease

For peer review only

outcomes – such as heart attacks, strokes, and blindness.[13] Unfortunately, although estimates of CVDRF prevalence from modelling studies exist, very little systematic, direct measurement of the burden of CVDRF in the country has occurred; although small outdated studies have suggested a high burden of CVDRF.[14-16 16-18] In sum, there is little rigorous information on the burden of CVDRF in Sierra Leone and no information on whether and how sufferers are accessing care. Sierra Leone is developing its national policy and strategic plan for NCDs. To ensure efficient use of the already stretched healthcare resources, the strategic plan and its implementation needs to be informed by empirical information on the burden of risk factors and current access to care.[19] In order to provide evidence to assist health policy planning, this study aimed to describe the prevalence of CVDRF in people over 40 years old in Sierra Leone, access to care for those risk factors, and sociodemographic characteristics associated with CVDRF and access to care.

METHODS

Study setting

The study was conducted in the district of Bo, located in the Southern Province of Sierra Leone, and one of 16 districts in the country. It has well documented rural and urban areas and contains Sierra Leone's second largest city, Bo.[20] The demographics, socio-economic circumstances, and geographical distribution of the population are similar to the larger Sierra-Leonean population.[20] In the last census in 2015 there were 575 478 inhabitants of Bo district with 66.1% (380 307) living in rural areas and 33.9% living in urban areas, mostly in Bo City. Up to 17.4% (100 188) of the population are over 40 years of age.[20] Bo District has a mainly agriculture-based economy, but service-based industries are growing. Mende is the most used language, but Krio and English are also spoken.

For peer review only

Sampling strategy

A sample size of 1893 participants was targeted to allow detection of diabetes prevalence (the risk factor thought likely to have the lowest prevalence) of 4% with a precision of ±1%. To allow for non-response and non-availability of data, we oversampled by 20%. A sampling of individuals over 40 years of age was done from rural or urban areas in proportion with known patterns from the 2015 populations and housing census of habitation of these areas in the over 40s.[20] Out of the 15 rural chiefdoms that comprise Bo District, seven chiefdoms, with widely dispersed communities were randomly chosen. Settlement groups or villages within these chiefdoms were identified and two were randomly chosen for study. Seven urban communities, randomly selected from 24 urban communities were also included in the study. Numbers of participants to sample from urban and rural areas were calculated based on the proportions of people living in these areas. In each urban community, numbers needed to study was 100. In each rural settlement or village, numbers needed to study was 93. If numbers were not achieved in the two selected areas, the next randomly ordered one was selected for study. Census information was not detailed enough to allow further identification of households with residents over 40 years old. Thus, data collection proceeded in each urban subdistrict or village, with data collectors starting at random points within each area and walking along a road or track sampling from every second household. Each household was permitted to enter no more than two people over 40 into the study. In villages where there were 93 households or fewer, all households were sampled. The geographical radius of the study was limited to 40 km from the centre of Bo to ensure accessibility. All chiefdoms and subdistricts in Bo were represented within this radius.

For peer review only

Data Collection

Data was collected electronically by trained staff using the ODK (Open Data Kit) platform.[21] from September-November 2018. The survey questionnaire was written in English but interviews were conducted in one of the local languages either Krio or Mende,

Survey questions asked about sociodemographic information - gender, age, highest level of education completed (no formal schooling, primary, junior secondary, senior secondary, higher education, or refused), employment the past 12 months (as government employee, non-government employee, self-employed, non-paid worker, student, homemaker, retired, unemployed able to work, unemployed unable to work, or refused), and marital status (as single, cohabiting, currently married, multiple partners, divorced, widowed, or refused). There were also 49 questions on household assets and construction materials. Questions on smoking, awareness of presence of CVDRF, and whether respondents were on treatment for these risk factors were based on the WHO Stepwise survey; for those who reported suffering from a CVDRF, or had had a stroke, heart attack, or angina, whether care had been accessed, where care was accessed, and reasons for not accessing care were also asked.

Height was measured using tape with participants standing with their backs, hips and heels against a wall and looking ahead horizontally (this method was validated using a Height Measure (SECA 213) during training). An Accuweight® digital body scale was used for measuring weight whilst wearing light clothing and without shoes.

Sitting blood pressure was measured using an Omron M6 AC LED Blood Pressure Monitor. Three measurements were taken with five minutes intervals between measurements. Blood samples were taken first thing in the morning after an 8 hour overnight fast. Glucose and

For peer review only

cholesterol were measured using the Accutrend® Plus Blood Test Meter (Diagnostics Roche) point of care device.

Participant's fasting status was checked prior to the blood sample being taken, and those who reported not fasting were labelled as such. Cholesterol samples were obtained from every second participant, while glucose was measured from all participants. The conversion rate of 1.11 was used to convert capillary glucose to plasma glucose.[22]

Outcome measures

Body mass index (BMI) was defined as weight (measured in kilograms (kg)) divided by height (measured in meters squared) and classified as normal weight (<25kg/m^2) or overweight/obese (BMI ≥ 25kg/m^2). An additional analysis with normal and overweight (<30kg/m^2) versus obese (BMI ≥ 30kg/m^2) was also done. Diabetes was defined as fasting plasma glucose (FPG) ≥7.0 mmol/L (126 mg/dL), or as random plasma glucose (RPG) ≥11.1 mmol/L (200 mg/dL). Hypertension was defined as recorded systolic blood pressure ≥ 140 or diastolic ≥ 90mmHg, calculated using the average of the final two readings. Dyslipidaemia was defined as measured total cholesterol level ≥ 6.21 mmol/L, or low-density lipoprotein (LDL) ≥ 4.1 mmol/L, or high-density lipoprotein (HDL) < 1.19 mmol/L. Participants that reported they had taken drugs for diabetes, hypertension or dyslipidaemia within the last two weeks were classified as having these conditions irrespective of their biomarker measurements. Smoking was defined as: current smoker if participants either reported currently smoking or had ceased within in the last year, or non-smoking for others. Educational level was defined as no completed education or any education. Marital status was defined as married/cohabiting or single/widowed/divorced. Wealth quintiles were derived from the first principal component of household assets and construction materials using the method of Filmer and Pritchett.[23]

For peer review only

Construction of the care cascade

A cascade of care was constructed for diabetes and hypertension. The stages in the care cascade are:

1) Prevalent disease (the population with hypertension or diabetes).

2) Ever been screened: Participants have had their blood pressure or blood glucose measured by a health care professional.

3) Prior diagnosis: Participants had ever been told by a doctor or other health care worker that they have hypertension or diabetes.

4) Currently on treatment: Participants who had taken drugs for hypertension or diabetes in the last two weeks.

5) Disease control: Participants who have their condition controlled to target at study measurement.

Entry into each subsequent stage of the cascade was contingent on an individual having achieved the previous stage. The population prevalence for diabetes and hypertension formed the denominators for all other stages of the respective care cascade. Additionally, the loss from each step in the care cascade was calculated using the people who had achieved the previous step as the denominator.

For peer review only

Statistical analysis

Statistical analysis was done using SPSS v24 (IBM, New York). Descriptive statistics were described using mean and SD for normally distributed continuous variables and median and IQR for non-normally distributed variables. Univariate associations between independent variables (demographic characteristics) and outcomes (CVDRFs) were tested using Chi Squared tests and Kendalls Tau-B for categorical variables and Mann-Whitney and Spearman's Rho for continuous variables. Multivariable analyses were performed using binary logistic regression with forced entry of all independent variables. For hypertension, factors associated with achieving each step in the cascade were tested. This was not done for diabetes as numbers were too small for meaningful results. A sensitivity analysis using BMI>30 as a cut off was done (appendix 1), and we decided to use age as categorical variable in the multivariable analysis due to non-linear association with some outcomes (for example demographic characteristics and CVDRF). Confidence intervals for proportions was calculated using according to a method described by Robert Newcombe derived from a procedure outlined by E.D Wilson.[24]

Probability weights for age and sex in Bo-South were calculated based upon the 2015 Population and Household Census.[20] All analyses were done using weight adjustments. Clustering at village level was adjusted for in the multivariable analyses.

Patient and public involvement statement

Participants were not directly involved in planning the study. Information was fed back to patients if they had abnormal measurements and they were referred to a local health care facility.

For peer review only

RESULTS

The final sample included 2071 individuals. The weighted demographic characteristics and prevalence of cardiovascular risk factors of the study population are presented in table 1. The unweighted proportions of demographic characteristics of participants with measured cholesterol versus not measured cholesterol are presented in appendix 2. Those who had their cholesterol measured were similar to those who did not. However, there were fewer males who had cholesterol measured.

Population characteristics and risk factor prevalence

The population predominately lived in rural areas (62.9%) and 49.0% of the study population was female. The median age was 51.0 years, 67.4% had not completed any education and 72.6% were married/cohabiting. The prevalence of hypertension was 49.6% (95% CI 44.1-44.7) whilst the prevalence of diabetes and dyslipidaemia were 3.5% (95% CI, 3.4-3.6) and 6.7% (95% CI, 6.5-7.0) respectively. Overweight or obesity (BMI $\geq$ 25kg/m^2) was present in 26.5% (95% CI, 26.3-26.8) of the study population and 25.6% (95% CI 25.4-25.9) of the participants were current or recent (within the last year) smokers. Altogether, 77.1% (95% CI, 76.6-77.5) of the study population had at least one CVDRF when including cholesterol (and limiting the denominator to those 789 who had cholesterol measured), whilst when excluding cholesterol as a variable (and with a denominator of 1896 who had information on all other CVDRF) the prevalence of at least one CVDRF was 74.5%. (95 CI, 74.3-74.8). Univariate associations between demographic characteristics and CVDRF are presented in appendix 3.

For peer review only

In the multivariable analysis (table 2) living in an urban area was independently associated with all CVDRFs except for dyslipidaemia (which was more prevalent in those living in rural areas). Male sex was independently associated with lower prevalence of CVDRF with the exceptions of smoking and the presence of any risk factor. Increasing age was independently associated with increasing prevalence of hypertension, diabetes or dyslipidaemia, and with a decreased prevalence of being overweight or smoking. The prevalence of CVDRFs according to age group and sex is shown in figure 1. Having any education compared to no complete education was independently associated with increased prevalence of all CVDRFs expect for smoking. Being married or cohabiting was independently associated with lower prevalence of all CVDRFs except for diabetes and obesity. Wealth remained independently associated with all CVDRFs except for smoking, where increasing wealth quintile was associated with a lower prevalence of smoking.

Access to healthcare

A total of 496 participants reported a previous diagnosis of hypertension, diabetes or dyslipidaemia, angina, heart attack or stroke. Of these, only 88 (17.74%) stated that they had accessed health care for their cardiovascular diseases in the last three months and only 8.87% had accessed health care in the last four weeks. The most common reasons for not accessing healthcare were thinking that it wasn't necessary (47.0%) or that it was too expensive (24.5%). Everyone who accessed care in the last three months visited a modern health facility, with 35.5% visiting community-based health service, and 63.2% a hospital-based health service. Nobody reported having visited a traditional healer for their condition.

For peer review only

The cascade of care for hypertension is shown in figure 2. Among those with hypertension, 59.2% reported that they had their blood pressure measured by a health care professional (screened), and 33.2% had ever been diagnosed with hypertension. There was a substantial loss to care at both steps, 40.8% and 44.0% respectively. Only 14.7% of people with hypertension were currently on treatment (taken medication for hypertension in the last two weeks), and of the people who were currently on treatment 31.2% achieved control. The last step of the cascade, being controlled, had the biggest loss to care from the previous step of 68.8%. In the multivariable analysis of the hypertension cascade, (table 3) people living in an urban area were significantly more likely to pass through all the steps of the cascade apart from being diagnosed. Women were more likely than men to be screened or diagnosed, but not treated; men were more likely than women to be controlled. There was no clear relationship between age groups and progress through the cascade. Having some education or being wealthier were significantly associated with passing through the first three steps of the cascade, but not with being controlled.

The cascade of care for diabetes is presented in figure 3. Out of all the people with diabetes in our study population (hyperglycaemic on measurement or taken medication in the last two weeks), the largest loss to care was at the stage of screening with only 57% of participants reporting that they had had their blood sugar measured at any time previously. There was a more modest loss to care for the next step with 32.9% of the participants with diabetes reporting that they had ever been told that they have diabetes. For the next step only 19% of the participants with diabetes reported that they had been taking treatment for diabetes in the last two weeks. Finally, 8.6% of the total population with diabetes had achieved control of their disease which is less than half the population that reported that they were on treatment. For diabetes the sample size was too small to do multivariable analysis with demographic characteristics in the different steps in the cascade.

For peer review only

DISCUSSION

This paper reports one of the first rigorously conducted studies to provide estimates of the prevalence of all CVDRFs in Sierra Leone; it is the first that we are aware of to publish on access to care for CVDRF. Our data suggest that the prevalence of CVDRFs in Sierra Leone is high with about 75% of the population over 40 having at least one CVDRF. The risk of having a CVDRF increased with age, and CVDRFs was more common in the urban population, among women, unmarried people and individuals with education and in the highest wealth quintile. Smoking was very common among men, giving them a higher overall risk of having at least one CVDRF. Also, our analysis revealed that there are very high rates of unmet need for hypertension and diabetes care. Less than 20% of the population with hypertension, diabetes and dyslipidaemia accessed health care in the last three months.

Although we sampled only one area in Sierra Leone, the population structure is similar to other areas in Sierra Leone except for Freetown.[20] Thus our findings give insight into the likely prevalence and associations across the country. Indeed, our estimate of hypertension of about 50% is similar to that found previously in Sierra Leone in the same age group in other areas.[14 15] There are very little data available on diabetes from Sierra Leone, but the most recent estimates, both empirical and modelled, were much higher than we found in our study.[17 18] For example the NCD Risk collaboration estimated prevalence of diabetes to be 7.1% (95% CI 3.5-12.1) in 2014.[18] The prevalence of diabetes in urban areas in our material (5.5%) was however similar to a previous study (6.2%) from 2012-2014 collected in only urban areas of Bo.[17] An older study conducted in Bo in 1997 reported a lower prevalence of 2.4% in the urban population and 0% in the rural population.[25] Diabetes prevalence might be rising with time, but the methodologies used in the previous studies makes comparisons

For peer review only

difficult. Both the previous studies were also much smaller in sample size (n=694 and n=501) than ours, and likely underpowered.

In contrast, the prevalence of hypertension in our study is higher than previous empirical data from the WHO STEPS survey conducted in 2009, and which found hypertension in 37% of males and 33% of females.[16] The population sampled in the previous WHO STEPS survey was younger (25-65 years) than in our study though, and the prevalence of hypertension is also likely to have increased in the past years.

Other areas in West-Africa have also reported a similarly high prevalence of CVDRFs to what we have found, although prevalence of hypertension in Sierra Leone in our study is higher than other regional estimates from countries like Nigeria and Ghana. [4 26-29] 25% of the population in our sample were overweight or obese which is surprising for one of the poorest countries in the world. However, our estimates of overweight/obesity are slightly lower than those derived from the WHO STEPS survey from 2009,[16] and lower than those reported from Nigeria, so it is unlikely that our findings over-estimate the prevalence.[28] The geographical, and socio-economic and education balance of most CVDRF that we found are also reflective of findings from other studies in the region. [28 29] However, in other studies, CVDRFs like diabetes and hypertension are more prevalent in males in contrast to our findings.[28 29] Still, overall, males actually have a higher risk of having at least one CVDRF than females in our sample. This makes males a vulnerable group when it comes CVDRFs, especially since the cascade analysis suggests that they are less likely to enter into the healthcare system for their conditions than women.

The low prevalence of people with hypertension being controlled for their condition is similar to what has been previously shown in countries in Sub-Saharan Africa.[4] Regarding diabetes, other studies have shown that many low-income countries in sub-Saharan Africa

perform better than Sierra Leone on access to care with an average of more than 15-20% of the patients achieving control of the disease.[5 30] However, similar to our findings, the biggest loss to care was at the stage of screening.[4] Although there are no studies done on the access to care for CVDRFs in Sierra Leone, previous studies on HIV-care has shown that the loss to care is substantial with only 22.8% of patients with newly diagnosed HIV receiving effective treatment.[31] It might be tenuous to compare HIV care and care for CVDRF, as HIV care receives substantial financial support from donors. Care for HIV is also largely separated from the public health care system, and health seeking behaviour for HIV is affected by stigma. Nevertheless, it is another indication that the health system in Sierra Leone finds it challenging to provide long-term follow up care for patients with chronic disorders.

Living in an urban area was a strong predictive factor for passing through the cascade steps and achieving control of hypertension. Women were more likely to be screened and diagnosed for hypertension than men which could be due to women accessing maternal and child health care (which has been a focus of healthcare efforts in Sierra Leone), gender norms, and facility opening hours. It is important to ensure that efforts are made to encourage and retain men in care. People with higher education and in the highest wealth quintile were also more likely to access care; similar to previous findings regarding access to hypertension care in LMICs.[4] Poorer and uneducated people are also more likely to experience catastrophic health expenditure on accessing care for non-communicable diseases,[32] and investments in improving hypertension care present an opportunity to reduce health inequalities between socioeconomic groups. Even if health care is free, which in Sierra Leone is the case for the 'destitute', Ebola survivors, pregnant women, lactating women, or children under 5,[33] accessing care still require transport costs and is time lost from income generating activity.[34] That we found that the most common reasons for not accessing care included cost suggests

For peer review only

that addressing this barrier is key to providing care for sufferers of CVDRF in Sierra Leone. Interestingly, the people most likely to access care in our study (high education and wealth) were less likely to succeed at the last step in the cascade by achieving control of their condition. One reason for this could be that medications are not taken regularly. However, this finding could also be due to lack of study power due to the low number of people reaching the last step in the cascade.

This study is one of the first studies to report prevalence of multiple CVDRFs in such a large sample from Sierra Leone and the first study to report access to care for these. The study sample is larger than any previous studies on CVDRF in Sierra Leone, and the data sampling and analysis were done in a rigorous way to avoid potential biases. Bo also consists of urban and rural areas that are similar to rest of Sierra Leone.[20] Hence the sample should be comparable to the rest of the population.

There are several limitations in this study. First of all, we could not measure cholesterol in the total population. However, appendix 1 shows that there were few differences between the populations with measured cholesterol versus those without cholesterol measurements. The data collection was also limited to within 40 km of Bo City due to accessibility from Bo and travel times. However, all chiefdoms were represented within this distance and were entered into the randomisation. It is unlikely that those areas further from Bo, as an urban centre, would be different to those not selected, as areas more than 40 km from Bo were close to other conurbations in neighbouring districts. We did not control for clustering at household level as few houses supplied more than one participant.

In this study we have showed that the prevalence of CVDRFs in one of poorest populations in the world is remarkably high, and the access to care is low. This should have major implications for health policy and planning in Sierra Leone in the years to come. Early deaths

For peer review only

and disability due to cardiovascular disease can disrupt the little economic development the country has experienced in recent years and should be given more attention. There is an urgent need to plan where appropriate interventions can be implemented in the most efficient way to make the most of the country's limited health care resources, in order to prevent CVDRFs and its consequences.

CONCLUSIONS

Sierra Leone is one of the poorest countries in the world with an underfunded health system that has been deprived of infrastructural development. The country is currently experiencing some economic growth, a decrease in maternal and paediatric mortality and an increase in NCDs similar to trends seen in other parts of the world. This study shows that about 75% of the population in Bo, Sierra Leone, has at least one cardiovascular risk factor and access to care is very low. In particular, men living in rural areas have a high cardiovascular risk profile and do not access care. This study fills a gap in knowledge that is needed to inform national plans for cardiovascular disease prevention and management.

Contributors JD, MDW, RN and IB conceived and designed the overall study. JD, TB, HW, RA and JL coordinated baseline data collection and preparation. JD, MDW, RN and IB contributed to the design of the household survey. MLO conducted the analysis, and wrote, and revised the manuscript. JD supervised the analysis, write up, and development of the manuscript. All authors substantively reviewed manuscripts, inputted into revisions, and approved the final manuscript.

Funding Support for the study was given by the Wellcome Trust.

Competing interest The authors report no competing interest in conducting this study.

Patient consent for publication Not required.

Ethical approval Ethical approval was sought and given from the Sierra Leone Ethical and Scientific Review Committee and the BDM Research Ethics sub-committee at King's College London (HR-17/18-7298). Consent to undertake the study was obtained from each village chief or community leader. Consent was obtained from all individuals participating in the study. In the event were participants were illiterate, the consent form was read out to them in the local language and an inked-thumb signature obtained.

Data availability statement No additional data available.

For peer review only

Acknowledgements
MDW acknowledges support from the NIHR Newcastle Biomedical Research Centre.
We thank the data collectors (DC) and field manager (FM) who worked on this study for their tireless commitment. These include: Ramatu Senesie DC; Allieu Abu Sheriff DC; Albert Sidikie Sama FM; Abdulai Kamara DC; Umu Binta Bah DC; Michael Dawson DC; Christiana Pratt DC; Michael E. Garrick DC; Peter Tamba Morsay DC; Francess Koker DC; Ismael Vandi DC; Samuel Kamanda DC; Wilfred A. U. Jimmy DC-Team Supervisor; Yvonne Vincent DC; Abu Bakarr Mansaray DC; Mariama Jalloh DC- Team Supervisor; In addition, we also want to thank and acknowledge the interns (Kadijatu Assiatu Kargbo; Amara Vandi Fomba; Rita kallon; Veronica Manty Marrah; Carpenter Emmanuel; Bangura A. Ronald; Kpallu Kpakila Sahr; Habibatu Adama Konuwa who supported our research team other research activities.

For peer review only

References

1 Roth GA, Forouzanfar MH, Moran AE, et al. Demographic and epidemiologic drivers of global cardiovascular mortality. *N Engl J Med* 2015;372(14):1333-41. doi: 10.1056/NEJMoa1406656
2 Collaborators GBDCoD. Global, regional, and national age-sex specific mortality for 264 causes of death, 1980-2016: a systematic analysis for the Global Burden of Disease Study 2016. *Lancet* 2017;390(10100):1151-210. doi: 10.1016/S0140-6736(17)32152-9
3 Lawes CM, Vander Hoorn S, Law MR, et al. Blood pressure and the global burden of disease 2000. Part II: estimates of attributable burden. *J Hypertens* 2006;24(3):423-30. doi: 10.1097/01.hjh.0000209973.67746.f0
4 Geldsetzer P, Manne-Goehler J, Marcus ME, et al. The state of hypertension care in 44 low-income and middle-income countries: a cross-sectional study of nationally representative individual-level data from 1.1 million adults. *Lancet* 2019;394(10199):652-62. doi: 10.1016/S0140-6736(19)30955-9
5 Manne-Goehler J, Geldsetzer P, Agoudavi K, et al. Health system performance for people with diabetes in 28 low- and middle-income countries: A cross-sectional study of nationally representative surveys. *PLoS Med* 2019;16(3):e1002751. doi: 10.1371/journal.pmed.1002751
6 Collaborators GBDCoD. Global, regional, and national age-sex-specific mortality for 282 causes of death in 195 countries and territories, 1980-2017: a systematic analysis for the Global Burden of Disease Study 2017. *Lancet* 2018;392(10159):1736-88. doi: 10.1016/S0140-6736(18)32203-7
7 The World Bank. Sierra Leone 2019 [Available from: https://data.worldbank.org/country/sierra-leone.
8 UNDP. Human Development Indices and Indicators: 2018 Statistical Update 2018 Available from: http://hdr.undp.org/sites/all/themes/hdr_theme/country-notes/SLE.pdf.
9 The World Bank. Mortality rate, under-5 (per 1,000 live births) - 2017. 2019 Available from: https://data.worldbank.org/indicator/SH.DYN.MORT.
10 The world Bank. Maternal mortality ratio (modeled estimate, per 100,000 live births) - 2017. 2019 Available from: The World Bank. Maternal mortality ratio (modeled estimate, per 100,000 live births) - 2017.
11 World Health Organisation. Ebola situation report 30 March. 1th edn. Geneva 2016 Available from: https://www.who.int/csr/disease/ebola/situation-reports/archive/en/.
12 Samba T, Bhat P, Owiti P, et al. Non-communicable diseases in the Western Area District, Sierra Leone, before and during the Ebola outbreak. *Public Health Action* 2017;7(Suppl 1):S16-S21. doi: 10.5588/pha.16.0086
13 Global Burden of Disease Study C. Global, regional, and national incidence, prevalence, and years lived with disability for 301 acute and chronic diseases and injuries in 188 countries, 1990-2013: a systematic analysis for the Global Burden of Disease Study 2013. *Lancet* 2015;386(9995):743-800. doi: 10.1016/S0140-6736(15)60692-4
14 Awad M, Ruzza A, Mirocha J, et al. Prevalence of hypertension in the Gambia and Sierra Leone, western Africa: a cross-sectional study. *Cardiovasc J Afr* 2014;25(6):269-78. doi: 10.5830/CVJA-2014-058
15 Meehan KA, Bankoski AJ, Tejan E, et al. Hypertension in Bo, Sierra Leone. *Ethn Dis* 2011;21(2):237-42
16 Tejan E, Meehan KA...
17 World Health Organisation. WHO STEPs Sierra Leone. 2009

18 Sundufu AJ, Bockarie CN, Jacobsen KH. The prevalence of type 2 diabetes in urban Bo, Sierra Leone, and in the 16 countries of the West Africa region. *Diabetes Metab Res Rev* 2017;33(7) doi: 10.1002/dmrr.2904

19 Collaboration NCDRF. Worldwide trends in diabetes since 1980: a pooled analysis of 751 population-based studies with 4.4 million participants. *Lancet* 2016;387(10027):1513-30. doi: 10.1016/S0140-6736(16)00618-8

20 World Health Organisation. National capacity to address and respond to NCDs: Existence of operational policies, strategies, or action plans 2016 Available from: http://www.who.int/gho/ncd/health_system_response/policy_text/en/.

21 Statistics Sierra Leone. 2015 Population and Housing Census 2015 Available from: https://www.statistics.sl/images/StatisticsSL/Documents/final-results_-2015_population_and_housing_census.pdf.

22 Hartung C. Open Data Kit: Tools to Build Information Services for Developing Regions 2010 [

23 D'Orazio P, Burnett RW, Fogh-Andersen N, et al. Approved IFCC recommendation on reporting results for blood glucose: International Federation of Clinical Chemistry and Laboratory Medicine Scientific Division, Working Group on Selective Electrodes and Point-of-Care Testing (IFCC-SD-WG-SEPOCT). *Clin Chem Lab Med* 2006;44(12):1486-90. doi: 10.1515/CCLM.2006.275

24 Filmer D, Pritchett LH. Estimating wealth effects without expenditure data--or tears: an application to educational enrollments in states of India. *Demography* 2001;38(1):115-32. doi: 10.1353/dem.2001.0003

25 Newcombe RG. Two-sided confidence intervals for the single proportion: comparison of seven methods. *Stat Med* 1998;17(8):857-72. doi: 10.1002/(sici)1097-0258(19980430)17:8<857::aid-sim777>3.0.co;2-e

26 Ceesay MM, Morgan MW, Kamanda MO, et al. Prevalence of diabetes in rural and urban populations in southern Sierra Leone: a preliminary survey. *Trop Med Int Health* 1997;2(3):272-7. doi: 10.1046/j.1365-3156.1997.d01-265.x

27 Cappuccio FP, Micah FB, Emmett L, et al. Prevalence, detection, management, and control of hypertension in Ashanti, West Africa. *Hypertension* 2004;43(5):1017-22. doi: 10.1161/01.HYP.0000126176.03319.d8

28 Ezejimofor M, Uthman O, Chen YF, et al. Magnitude and pattern of hypertension in the Niger Delta: a systematic review and meta-analysis of community-based studies. *J Glob Health* 2018;8(1):010420. doi: 10.7189/jogh.08.010420

29 Oguoma VM, Nwose EU, Skinner TC, et al. Prevalence of cardiovascular disease risk factors among a Nigerian adult population: relationship with income level and accessibility to CVD risks screening. *BMC Public Health* 2015;15:397. doi: 10.1186/s12889-015-1709-2

30 Kodaman N, Aldrich MC, Sobota R, et al. Cardiovascular Disease Risk Factors in Ghana during the Rural-to-Urban Transition: A Cross-Sectional Study. *PLoS One* 2016;11(10):e0162753. doi: 10.1371/journal.pone.0162753

31 Price AJ, Crampin AC, Amberbir A, et al. Prevalence of obesity, hypertension, and diabetes, and cascade of care in sub-Saharan Africa: a cross-sectional, population-based study in rural and urban Malawi. *Lancet Diabetes Endocrinol* 2018;6(3):208-22. doi: 10.1016/S2213-8587(17)30432-1

32 Kelly JD, Schlough GW, Conteh S, et al. The Majority of the Pre-Antiretroviral Population Who Were Lost to Follow-Up Stopped Their Care in Freetown, Sierra Leone: A 12-Month Prospective Cohort Study Starting with HIV Diagnosis. *PLoS One* 2016;11(2):e0149584. doi: 10.1371/journal.pone.0149584

33 Jan S, Laba TL, Essue BM, et al. Action to address the household economic burden of non-communicable diseases. *Lancet* 2018;391(10134):2047-58. doi: 10.1016/S0140-6736(18)30323-4

34 Treacy L, Bolkan HA, Sagbakken M. Distance, accessibility and costs. Decision-making during childbirth in rural Sierra Leone: A qualitative study. *PLoS One* 2018;13(2):e0188280. doi: 10.1371/journal.pone.0188280

35 Chimbindi N, Bor J, Newell ML, et al. Time and Money: The True Costs of Health Care Utilization for Patients Receiving "Free" HIV/Tuberculosis Care and Treatment in Rural KwaZulu-Natal. *J Acquir Immune Defic Syndr* 2015;70(2):e52-60. doi: 10.1097/QAI.0000000000000728

For peer review only

Table 1 Weighted demographic characteristics and prevalence of cardiovascular risk factors in Bo, Sierra Leone (n=2071)

parameter	group	Proportions using weights
Place of living	Rural	62.90%
	Urban	37.10%
Gender	Female	49.00%
	Male	51.00%
Age median (IQR) n = 2062		51.0 (45.0-63.0)
education level n = 2070	No completed education	67.40%
	Any education	32.60%
marital status n = 2069	Married/Cohabiting	72.60%
	Single/widowed/divorced	27.40%
wealth quintile n = 1991	1	20.50%
	2	20.50%
	3	20.00%
	4	19.90%
	5	19.10%
Cardiovascular disease risck factors (CVDRF)	Hypertension n =2070	49.60%
	Mean (SD) SBP	136.19 (25.24)
	Mean (SD) DBP	87.52 (14.11)
	Diabetes n = 2019	3.50%
	Dyslipidaemia n = 840	6.70%
	Overweight/obesity n = 1947	26.50%
	Smoking	25.60%
	One CVD risk factor or more out of a possible 7 - including cholesterol (n = 789)	Including cholesterol<br><br>77.10%
	One CVD risk factor or more out of a possible 6 - excluding cholesterol (n = 1896)	Excluding cholesterol<br><br>74.50%

For peer review only

Table 2 Multivariable associations between demographic characteristics and cardiovascular risk factors including cholesterol (n=2071)

parameter	group	Hypertension		Diabetes		Dyslipidaemia		Overweight/obese		Smoking		Total CVD risk factors incl. chol		Total CVD risk factors exl. chol	
		OR (95% CI)	P-value	OR (95% CI)	P-value	OR (95% CI)	P-value	OR (95% CI)	P-value	OR (95% CI)	P-value	OR (95% CI)	P-value	OR (95% CI)	P-value
Place of living	Rural	Referent	-	Referent	-	Referent	-	Referent	-	Referent	-	Referent	-	Referent	-
	Urban	1.04 (1.01-1.08)	0.014	1.46 (1.34-1.60)	<0.001	0.84 (0.75-0.93)	0.001	1.17 (1.12-1.21)	<0.001	1.13 (1.08-1.17)	<0.001	0.99 (0.93-1.05)	0.614	1.06 (1.02-1.10)	0.002
Gender	Female	Referent	-	Referent	-	Referent	-	Referent	-	Referent	-	Referent	-	Referent	-
	Male	0.78 (0.75-0.80)	<0.001	0.75 (0.69-0.82)	<0.001	0.88 (0.80-0.97)	0.013	0.31 (0.30-0.32)	<0.001	9.15 (8.76-9.54)	<0.001	1.6 (1.52-1.70)	<0.001	1.43 (1.38-1.48)	<0.001
Age	40-49	Referent	-	Referent	-	Referent	-	Referent	-	Referent	-	Referent	-	Referent	-
	50-59	1.75 (1.69-1.81)	<0.001	2.10 (1.91-2.32)	<0.001	1.38 (1.25-1.53)	<0.001	0.84 (0.81-0.87)	<0.001	0.84 (0.81-0.88.0)	<0.001	0.93 (0.88-0.99)	0.023	1.15 (1.11-1.20)	<0.001
	60-69	2.35 (2.26-2.45)	<0.001	2.77 (2.50-3.07)	<0.001	1.36 (1.22-1.53)	<0.001	0.85 (0.81-0.89)	<0.001	0.58 (0.56-0.61)	<0.001	1.25 (1.16-1.35)	<0.001	1.70 (1.60-1.81)	<0.001
	70-79	3.43 (3.27-3.61)	<0.001	3.46 (3.07-3.89)	<0.001	1.76 (1.52-2.05)	<0.001	0.70 (0.66-0.75)	<0.001	0.36 (0.33-0.38)	<0.001	2.24 (2.00-2.51)	<0.001	1.25 (1.16-1.34)	<0.001
	>80	3.13 (2.96-3.32)	<0.001	1.76 (1.69-1.99)	<0.001	0.98 (0.81-1.19)	0.835	0.49 (0.45-0.53)	<0.001	0.52 (0.48-0.56)	<0.001	1.22 (1.09-1.38)	0.001	1.07 (1.16-1.34)	<0.001
Education level	No complete education	Referent	-	Referent	-	Referent	-	Referent	-	Referent		Referent		Referent	-
	Any education	1.17 (1.14-1.21)	<0.001	1.83 (1.69-1.99)	<0.001	1.08 (0.98-1.18)	0.111	1.63 (1.57-1.69)	<0.001	0.86 (0.83-0.89)	<0.001	0.91 (0.86-0.96)	0.001	1.07 (1.04-1.11)	<0.001
Marital status	Single/divorced/widow	Referent	-	Referent	-	Referent	-	Referent	-	Referent	-	Referent	-	Referent	-
	Married/Cohabiting	0.8 (0.78-0.83)	<0.001	1.01 (0.93-1.11)	0.785	0.62 (0.56-0.68)	<0.001	1.1 (1.06-1.15)	<0.001	0.8 (0.77-0.84)	<0.001	0.81 (0.76-0.86)	<0.001	0.84 (0.81-0.88)	<0.001

For peer review only - http://bmjopen.bmj.com/site/about/guidelines.xhtml

Wealth quintile	1	Referent	-	Referent	-	Referent	-	Referent	-	Referent	-	Referent	-	Referent	-
	2	0.83 (0.79-0.86)	<0.001	1.31 (1.14-1.52)	<0.001	1.84 (1.47-2.29)	<0.001	1.42 (1.34-1.50)	<0.001	0.96 (0.92-1.01)	0.105	1.06 (0.98-1.14)	0.142	1.1 (1.05-1.15)	<0.001
	3	0.99 (0.95-1.03)	0.698	1.2 (1.04-1.39)	0.014	2.36 (1.90-2.29)	<0.001	1.66 (1.57-1.76)	<0.001	0.71 (0.68-0.75)	<0.001	0.87 (0.81-0.94)	0.001	0.91 (0.87-0.95)	0.001
	4	1.3 (1.25-1.36)	<0.001	1.64 (1.42-1.88)	<0.001	6.19 (5.07-7.56)	<0.001	3 (2.84-3.16)	<0.001	0.51 (0.49-0.54)	<0.001	1.41 (1.30-1.53)	<0.001	1.32 (1.25-1.38)	<0.001
	5	1.6 (1.52-1.69)	<0.001	2.7 (2.34-3.12)	<0.001	11.16 (9.05-13.76)	<0.001	5.11 (4.81-5.44)	<0.001	0.39 (0.37-0.42)	<0.001	2.46 (2.23-2.73)	<0.001	1.62 (1.53-1.72)	<0.001

For peer review only

Table 3 Multivariate associations between demographic characteristics and access to care for hypertension for people with hypertension (n=1092)

parameter	group	Screened (n=1092)		Diagnosis (n=646)		Treated (n=362)		Controlled (n=160)	
		OR (95% CI)	P-value	OR (95% CI)	P-value	OR (95% CI)	P-value	OR (95% CI)	P-value
Place of living	Rural	Referent	-	Referent	-	Referent	-	Referent	-
	Urban	1.61 (1.53-1.68)	<0.001	0.97 (0.91-1.03)	0.325	1.36 (1.23-1.50)	<0.001	2.13 (1.77-2.58)	<0.001
Gender	Female	Referent	-	Referent	-	Referent	-	Referent	-
	Male	0.70 (0.67-0.73)	<0.001	0.79 (0.74-0.84)	<0.001	1.13 (1.02-1.25)	0.015	1.12 (0.97-1.30)	0.121
Age	40-49	Referent	-	Referent	-	Referent	-	Referent	-
	50-59	0.91 (0.87-0.96)	0.001	1.36 (1.28-1.45)	<0.001	1.64 (1.49-1.80)	<0.001	0.95 (0.82-1.11)	0.535
	60-69	1.49 (1.41-1.58)	<0.001	1.01 (0.94-1.07)	0.874	2.15 (1.93-2.39)	<0.001	0.89 (0.76-1.04)	0.141
	70-79	1.22 (1.14-1.30)	<0.001	0.55 (0.51-0.60)	<0.001	1.44 (1.25-1.65)	<0.001	1.13 (0.93-1.38)	0.221
	>80	0.63 (0.59-0.68)	<0.001	0.72 (0.64-0.80)	<0.001	1.55 (1.29-1.87)	<0.001	1.09 (0.84-1.42)	0.516
Education level	No complete education	Referent	-	Referent	-	Referent	-	Referent	-
	Any education	1.78 (1.69-1.86)	<0.001	1.09 (1.03-1.16)	0.002	2.93 (2.69-3.18)	<0.001	0.70 (0.62-0.80)	<0.001
Marital status	Single/divorced/widow	Referent	-	Referent	-	Referent	-	Referent	-
	Married/Cohabiting	1.02 (0.97-1.07)	0.465	0.95 (0.89-1.01)	0.12	0.67 (0.61-0.73)	<0.001	1.17 (1.02-1.33)	0.022
Wealth quintile	1	Referent	-	Referent	-	Referent	-	Referent	-
	2	1.22 (1.15-1.30)	<0.001	1.59 (1.45-1.75)	<0.001	2.40 (1.97-2.91)	<0.001	0.25 (0.18-0.37)	<0.001
	3	1.77 (1.67-1.88)	<0.001	1.63 (1.50-1.79)	<0.001	2.95 (2.47-3.53)	<0.001	0.17 (0.12-0.22)	<0.001
	4	2.63 (2.47-2.80)	<0.001	1.45 (1.33-1.58)	<0.001	5.37 (4.52-6.39)	<0.001	0.16 (0.12-0.22)	<0.001
	5	4.21 (3.91-4.54)	<0.001	2.24 (2.04-2.46)	<0.001	4.97 (4.16-5.93)	<0.001	0.28 (0.20-0.39)	<0.001

For peer review only

Caption Figure 1: Prevalence of cardiovascular risk factors (CVDRF) according to age group and gender

Caption Figure 2: The cascade of care for hypertension

Caption Figure 3: The cascade of care diabetes

For peer review only

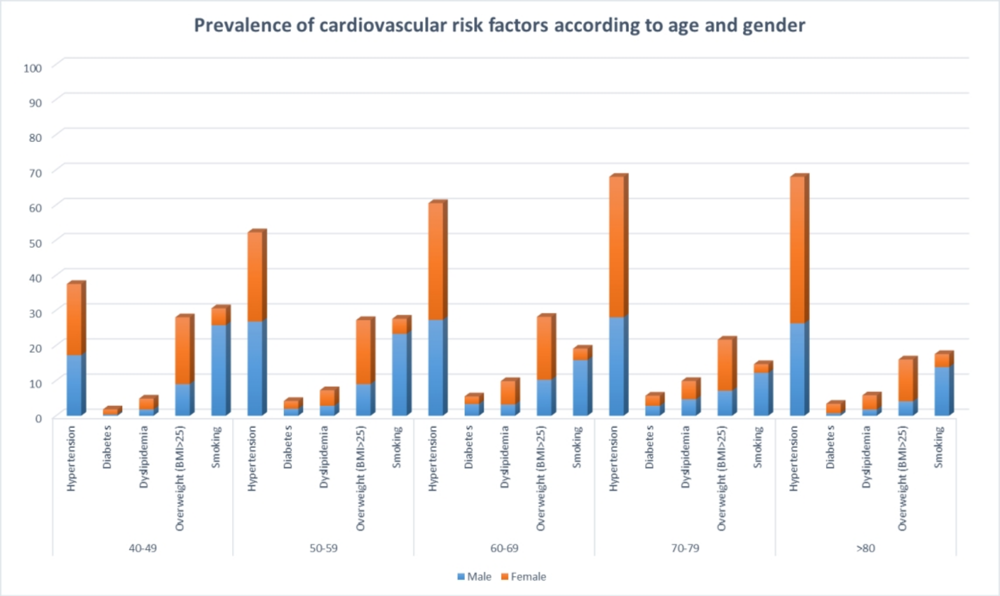

Prevalence of cardiovascular risk factors (CVDRF) according to age group and gender

263x156mm (150 x 150 DPI)

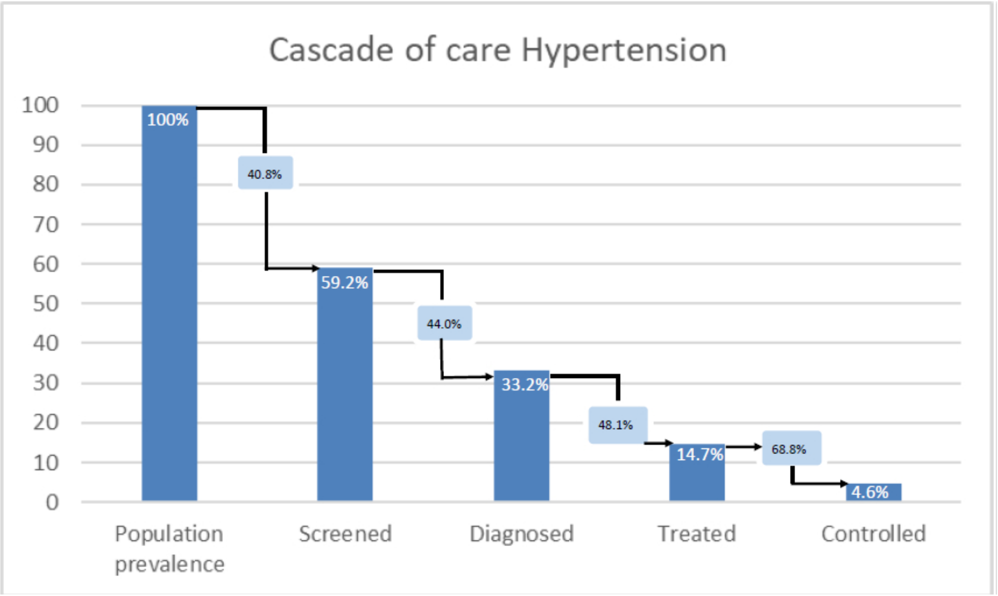

The cascade of care for hypertension

158x94mm (144 x 144 DPI)

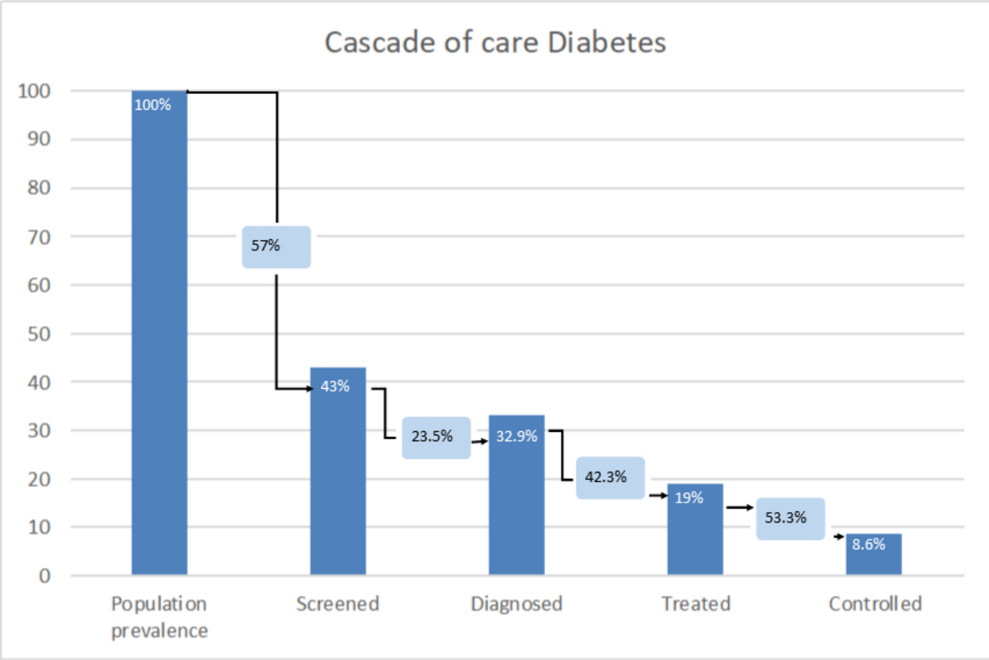

The cascade of care diabetes

Appendix table 1 Sensitivity analysis with BMI >30 of multivariable associations between demographic characteristics and cardiovascular risk (n=2071)

parameter	group	Hypertension		Diabetes		Dyslipidaemia		Obesity (BMI>30)		Smoking		Total CVD risk factors incl. chol		Total CVD risk factors exl. chol	
		OR (95% CI)	P-value	OR (95% CI)	P-value	OR (95% CI)	P-value	OR (95% CI)	P-value	OR (95% CI)	P-value	OR (95% CI)	P-value	OR (95% CI)	P-value
Place of living	Rural	Referent	-	Referent	-	Referent	-	Referent	-	Referent	-	Referent	-	Referent	-
	Urban	1.04 (1.01-1.08)	0.014	1.46 (1.34-1.60)	<0.001	0.84 (0.75-0.93)	0.001	1.4 (1.33-1.48)	<0.001	1.13 (1.08-1.17)	<0.001	0.99 (0.93-1.05)	0.614	1.06 (1.02-1.10)	0.002
Gender	Female	Referent	-	Referent	-	Referent	-	Referent	-	Referent	-	Referent	-	Referent	-
	Male	0.78 (0.75-0.80)	<0.001	0.75 (0.69-0.82)	<0.001	0.88 (0.80-0.97)	0.013	0.24 (0.23-0.26)	<0.001	9.15 (8.76-9.54)	<0.001	1.6 (1.52-1.70)	<0.001	1.43 (1.38-1.48)	<0.001
Age	40-49	Referent	-	Referent	-	Referent	-	Referent	-	Referent	-	Referent	-	Referent	-
	50-59	1.75 (1.69-1.81)	<0.001	2.10 (1.91-2.32)	<0.001	1.38 (1.25-1.53)	<0.001	0.74 (0.70-0.79)	<0.001	0.84 (0.81-0.880	<0.001	0.93 (0.88-0.99)	0.023	1.15 (1.11-1.20)	<0.001
	60-69	2.35 (2.26-2.45)	<0.001	2.77 (2.50-3.07)	<0.001	1.36 (1.22-1.53)	<0.001	0.85 (0.80-0.91)	<0.001	0.58 (0.56-0.61)	<0.001	1.25 (1.16-1.35)	<0.001	1.70 (1.60-1.81)	<0.001
	70-79	3.43 (3.27-3.61)	<0.001	3.46 (3.07-3.89)	<0.001	1.76 (1.52-2.05)	<0.001	0.64 (0.59-0.70)	<0.001	0.36 (0.33-0.38)	<0.001	2.24 (2.00-2.51)	<0.001	1.25 (1.16-1.34)	<0.001
	>80	3.13 (2.96-3.32)	<0.001	1.76 (1.69-1.99)	<0.001	0.98 (0.81-1.19)	0.835	0.59 (0.53-0.66)	<0.001	0.52 (0.48-0.56)	<0.001	1.22 (1.09-1.38)	0.001	1.07 (1.16-1.34)	<0.001
Education level	No complete education	Referent	-	Referent	-	Referent	-	Referent	-	Referent		Referent		Referent	-
	Any education	1.17 (1.14-1.21)	<0.001	1.83 (1.69-1.99)	<0.001	1.08 (0.98-1.18)	0.111	1.26 (1.20-1.33)	<0.001	0.86 (0.83-0.89)	<0.001	0.91 (0.86-0.96)	0.001	1.07 (1.04-1.11)	<0.001
Marital status	Single/divorced/widow	Referent	-	Referent	-	Referent	-	Referent	-	Referent	-	Referent	-	Referent	-
	Married/Cohabiting	0.8 (0.78-0.83)	<0.001	1.01 (0.93-1.11)	0.785	0.62 (0.56-0.68)	<0.001	1.26 (1.20-1.33)	<0.001	0.8 (0.77-0.84)	<0.001	0.81 (0.76-0.86)	<0.001	0.84 (0.81-0.88)	<0.001

For peer review only

Wealth quintile	1	Referent	-	Referent	-	Referent	-	Referent	-	Referent	-	Referent	-	Referent	-	Referent	-
	2	0.83 (0.79-0.86)	<0.001	1.31 (1.14-1.52)	<0.001	1.84 (1.47-2.29)	<0.001	2 (1.83-2.20)	<0.001	0.96 (0.92-1.01)	0.105	1.06 (0.98-1.14)	0.142	1.1 (1.05-1.15)	<0.001
	3	0.99 (0.95-1.03)	0.698	1.2 (1.04-1.39)	0.014	2.36 (1.90-2.29)	<0.001	2.08 (1.90-2.78)	<0.001	0.71 (0.68-0.75)	<0.001	0.87 (0.81-0.94)	0.001	0.91 (0.87-0.95)	0.001
	4	1.3 (1.25-1.36)	<0.001	1.64 (1.42-1.88)	<0.001	6.19 (5.07-7.56)	<0.001	3.26 (2.98-3.55)	<0.001	0.51 (0.49-0.54)	<0.001	1.41 (1.30-1.53)	<0.001	1.32 (1.25-1.38)	<0.001
	5	1.6 (1.52-1.69)	<0.001	2.7 (2.34-3.12)	<0.001	11.16 (9.05-13.76)	<0.001	4.5 (4.12-4.93)	<0.001	0.39 (0.37-0.42)	<0.001	2.46 (2.23-2.73)	<0.001	1.62 (1.53-1.72)	<0.001

For peer review only

For peer review only

Appendix table 2 Demographic characteristics of participants with and without measured cholesterol (n = 2071)

parameter	group	Not measured cholesterol	Measured Cholesterol
Place of living	Rural	61.30%	61.50%
	Urban	38.70%	38.50%
Gender	Male	47.30%	40.60%
	Female	52.70%	59.4%*
Age median (IQR)		56 (46-67)	55 (47-68)
Education level	No completed education	70.10%	67.50%
	Any education	29.90%	32.30%
Marital status	Married/Cohabiting	69.20%	67.90%
	Single/widowed/divorced	30.80%	32.10%
Wealth quintile	1	21.10%	18.20%
	2	20.10%	19.80%
	3	20.80%	19.00%
	4	17.90%	23.10%
	5	20.10%	19.80%

*p<0.005

For peer review only

Appendix table 3 Univariable associations between demographic characteristics and cardiovascular risk factors (n=2071)

parameter	group	Hypertension	Diabetes	Hypercholesterolaemia	Obesity	Smoking	One CVD risk factor or incl. Cholesterol	One CVD risk factors or more exc. Cholesterol
Place of living	Rural	46.00%	2.30%	5.00%	20.40%	28.40%	75.50%	72.60%
	Urban	55.8%*	5.5%*	9.6%*	37.2%*	21.0%*	79.6%*	77.8%*
Gender	Female	54.80%	4.00%	7.90%	36.70%	8.40%	74.80%	72.30%
	Male	44.7%*	3.0%*	5.3%*	17.0%*	42.2%*	79.5%*	76.7%*
Age		0.239*	0.081*	0.080*	-0.039*	-0.114*	0.068*	0.073*
Age groups	40-49	37.50%	1.80%	4.90%	28.00%	30.60%	75.00%	71.60%
	50-59	52.20%	4.20%	7.30%	27.20%	27.60%	75.40%	75.70%
	60-69	60.50%	5.70%	9.80%	28.20%	19.10%	80.60%	76.30%
	70-79	68.00%	5.70%	9.90%	21.70%	14.70%	84.40%	80.40%
	>80	68.0%**	3.4%**	5.8%**	16.0%**	17.6%**	80.6%**	77.2%**
Education level	No completed education	48.50%	2.60%	5.60%	22.80%	24.60%	76.00%	72.90%
	Any education	52.0%*	5.4%*	8.9%*	34.3%*	27.8%*	79.1%*	77.7%*
Marital status	Married/Cohabiting	45.50%	3.20%	5.70%	25.50%	29.20%	76.30%	73.70%
	Single/divorced/ Widowed	60.6%*	4.3%*	9.4%*	29.4%*	16.1%*	79.1%*	76.7%*
Wealth quintile	1	45.80%	1.70%	1.60%	13.80%	32.00%	73.40%	71.10%
	2	41.80%	2.30%	3.10%	17.50%	32.10%	75.70%	73.40%
	3	46.90%	2.40%	3.80%	21.50%	26.60%	71.50%	70.20%
	4	54.00%	3.70%	9.50%	34.60%	20.40%	79.10%	77.50%
	5	62.4%**	7.7%**	15.0%**	50.6%**	16.0%**	86.3%**	82.2%**

*P<0.001, **P for trend<0.001

For peer review only

STROBE Statement—Checklist of items that should be included in reports of ***cross-sectional studies***

	Item No	Recommendation
**Title and abstract**	1	(*a*) Indicate the study's design with a commonly used term in the title or the abstract
		(*b*) Provide in the abstract an informative and balanced summary of what was done and what was found
**Introduction**		
Background/rationale	2	Explain the scientific background and rationale for the investigation being reported
Objectives	3	State specific objectives, including any prespecified hypotheses
**Methods**		
Study design	4	Present key elements of study design early in the paper
Setting	5	Describe the setting, locations, and relevant dates, including periods of recruitment, exposure, follow-up, and data collection
Participants	6	(*a*) Give the eligibility criteria, and the sources and methods of selection of participants
Variables	7	Clearly define all outcomes, exposures, predictors, potential confounders, and effect modifiers. Give diagnostic criteria, if applicable
Data sources/ measurement	8*	For each variable of interest, give sources of data and details of methods of assessment (measurement). Describe comparability of assessment methods if there is more than one group
Bias	9	Describe any efforts to address potential sources of bias
Study size	10	Explain how the study size was arrived at
Quantitative variables	11	Explain how quantitative variables were handled in the analyses. If applicable, describe which groupings were chosen and why
Statistical methods	12	(*a*) Describe all statistical methods, including those used to control for confounding
		(*b*) Describe any methods used to examine subgroups and interactions
		(*c*) Explain how missing data were addressed
		(*d*) If applicable, describe analytical methods taking account of sampling strategy
		(*e*) Describe any sensitivity analyses
**Results**		
Participants	13*	(a) Report numbers of individuals at each stage of study—eg numbers potentially eligible, examined for eligibility, confirmed eligible, included in the study, completing follow-up, and analysed
		(b) Give reasons for non-participation at each stage
		(c) Consider use of a flow diagram
Descriptive data	14*	(a) Give characteristics of study participants (eg demographic, clinical, social) and information on exposures and potential confounders
		(b) Indicate number of participants with missing data for each variable of interest
Outcome data	15*	Report numbers of outcome events or summary measures
Main results	16	(*a*) Give unadjusted estimates and, if applicable, confounder-adjusted estimates and their precision (eg, 95% confidence interval). Make clear which confounders were adjusted for and why they were included
		(*b*) Report category boundaries when continuous variables were categorized
		(*c*) If relevant, consider translating estimates of relative risk into absolute risk for a meaningful time period
Other analyses	17	Report other analyses done—eg analyses of subgroups and interactions, and sensitivity analyses

For peer review only - http://bmjopen.bmj.com/site/about/guidelines.xhtml

**Discussion**		
Key results	18	Summarise key results with reference to study objectives
Limitations	19	Discuss limitations of the study, taking into account sources of potential bias or imprecision. Discuss both direction and magnitude of any potential bias
Interpretation	20	Give a cautious overall interpretation of results considering objectives, limitations, multiplicity of analyses, results from similar studies, and other relevant evidence
Generalisability	21	Discuss the generalisability (external validity) of the study results
**Other information**		
Funding	22	Give the source of funding and the role of the funders for the present study and, if applicable, for the original study on which the present article is based

*Give information separately for exposed and unexposed groups.

Note: An Explanation and Elaboration article discusses each checklist item and gives methodological background and published examples of transparent reporting. The STROBE checklist is best used in conjunction with this article (freely available on the Web sites of PLoS Medicine at http://www.plosmedicine.org/, Annals of Internal Medicine at http://www.annals.org/, and Epidemiology at http://www.epidem.com/). Information on the STROBE Initiative is available at www.strobe-statement.org.

For peer review only

BMJ Open

Prevalence and access to care for cardiovascular risk factors in older people in Sierra Leone: A cross-sectional survey

Journal:	*BMJ Open*
Manuscript ID	bmjopen-2020-038520.R1
Article Type:	Original research
Date Submitted by the Author:	06-Jul-2020
Complete List of Authors:	Odland, Maria Lisa; University of Birmingham Institute of Applied Health Research<br>Bockarie, Tahir; University of Warwick Warwick Medical School<br>Wurie, Haja; University of Sierra Leone College of Medicine and Allied Health Sciences<br>Ansumana, Rashid; Mercy Hospital Research Laboratory<br>Lamin, Joseph; Mercy Hospital Research Laboratory<br>Nugent, Rachel; RTI International<br>Bakolis, Ioannis; King's College London, London, United Kingdom, Centre for Implementation Science, Health Services and Population Research Department, Institute of Psychiatry, Psychology and Neuroscience, ; Department of Biostatistics and Health Informatics, Institute of Psychiatry, Psychology and Neuroscience, King's College London, London, United Kingdom<br>Witham, Miles; AGE Research Group, NIHR Newcastle Biomedical Research Centre, Newcastle University; Newcastle Upon Tyne Hospitals NHS Foundation Trust<br>Davies, Justine ; University of Birmingham Institute of Applied Health Research; King's College London, Centre for Global Health
**Primary Subject Heading**:	Global health
Secondary Subject Heading:	Cardiovascular medicine, Epidemiology, Diabetes and endocrinology, Global health, Public health
Keywords:	Health policy < HEALTH SERVICES ADMINISTRATION & MANAGEMENT, Quality in health care < HEALTH SERVICES ADMINISTRATION & MANAGEMENT, Organisation of health services < HEALTH SERVICES ADMINISTRATION & MANAGEMENT, EPIDEMIOLOGY, PUBLIC HEALTH

SCHOLARONE™
Manuscripts

I, the Submitting Author has the right to grant and does grant on behalf of all authors of the Work (as defined in the below author licence), an exclusive licence and/or a non-exclusive licence for contributions from authors who are: i) UK Crown employees; ii) where BMJ has agreed a CC-BY licence shall apply, and/or iii) in accordance with the terms applicable for US Federal Government officers or employees acting as part of their official duties; on a worldwide, perpetual, irrevocable, royalty-free basis to BMJ Publishing Group Ltd ("BMJ") its licensees and where the relevant Journal is co-owned by BMJ to the co-owners of the Journal, to publish the Work in this journal and any other BMJ products and to exploit all rights, as set out in our licence.

The Submitting Author accepts and understands that any supply made under these terms is made by BMJ to the Submitting Author unless you are acting as an employee on behalf of your employer or a postgraduate student of an affiliated institution which is paying any applicable article publishing charge ("APC") for Open Access articles. Where the Submitting Author wishes to make the Work available on an Open Access basis (and intends to pay the relevant APC), the terms of reuse of such Open Access shall be governed by a Creative Commons licence – details of these licences and which Creative Commons licence will apply to this Work are set out in our licence referred to above.

Other than as permitted in any relevant BMJ Author's Self Archiving Policies, I confirm this Work has not been accepted for publication elsewhere, is not being considered for publication elsewhere and does not duplicate material already published. I confirm all authors consent to publication of this Work and authorise the granting of this licence.

For peer review only

Prevalence and access to care for cardiovascular risk factors in older people in Sierra Leone: A cross-sectional survey

Maria Lisa Odland,[1*] Tahir Bockarie,[2] Haja Wurie,[3] Rashid Ansumana,[4] Joseph Lamin,[4] Rachel Nugent,[5] Ioannis Bakolis,[6,7] Miles Witham[8,9] and Justine Davies[1,9,10]

[1] University of Birmingham Institute of Applied Health Research, University of Birmingham, Birmingham, West Midlands, UK

[2] University of Warwick, Warwick Medical School, Coventry, UK

[3] University of Sierra Leone College of Medicine and Allied Health Sciences, Freetown, Western Area, SL

[4] Mercy Hospital Research Laboratory, Freetown, Sierra Leone

[5] RTI International, Seattle, WA, USA

[6] King's College London, London, United Kingdom, Centre for Implementation Science, Health Services and Population Research Department, Institute of Psychiatry, Psychology and Neuroscience, London, London, UK

[7] Department of Biostatistics and Health Informatics, Institute of Psychiatry, Psychology and Neuroscience, King's College London, London, United Kingdom London, UK

[8] AGE Research Group, NIHR Newcastle Biomedical Research Centre, Newcastle University Newcastle upon Tyne, UK

[9] Newcastle Upon Tyne Hospitals NHS Foundation Trust Newcastle Upon Tyne, Newcastle upon Tyne, UK

[10] King's College London, Centre for Global Health, London, UK

*Corresponding author

Maria Lisa Odland

Email: m.l.odland@bham.ac.uk

Word count: 4576 excluding title page, strengths and limitations, abstract and references.

For peer review only - http://bmjopen.bmj.com/site/about/guidelines.xhtml

Strengths and limitations of this study

- This study was adequately powered to detect cardiovascular risk factors in this population.
- We used random sampling and probability weights to avoid potential biases.
- The data collection was limited to one district in Sierra Leone.
- We did not control for clustering at household level as few houses supplied more than one participant.
- Clinical diagnoses in this study were defined for the purpose of this study based on measurements taken at a single point in time.

For peer review only

For peer review only - http://bmjopen.bmj.com/site/about/guidelines.xhtml

ABSTRACT

Introduction Prevalence of cardiovascular disease risk factors (CVDRF) is increasing, especially in low-income countries. In Sierra Leone, there is limited empirical data on the prevalence of CVDRFs, and there are no previous studies on the access to care for these conditions.

Methods This study in rural and urban Sierra Leone collected demographic, anthropometric measurements, and clinical data from randomly sampled individuals over 40 years old using a household survey. We describe prevalence of the following risk factors; diabetes, hypertension, dyslipidaemia, overweight or obesity, smoking and having at least one of these risk factors. Cascades of care were constructed for diabetes and hypertension, using % of the population with the disease who had previously been tested ("screened"), knew of their condition ("diagnosed), were on treatment ("treated"), or were controlled to target ("controlled"). Multivariable regression was used to test associations between prevalence of CVDRFs and progress through the cascade for hypertension with demographic and socioeconomic variables. In those with recognised disease who did not seek care reasons for not accessing care were recorded.

Results Of 2071 people, 49.6% (95% CI 49.3-50.0) of the population had hypertension, 3.5% (3.4-3.6) had diabetes, 6.7% (6.5-7.0) had dyslipidaemia, 25.6% (25.4-25.9) smoked, and 26.5% (26.3-26.8%) were overweight/obese; a total of 77.1% (76.6-77.5%) had at least one CVDRF. People in urban areas were more likely to have diabetes and be overweight than those living in rural areas. Moreover, being female, more educated, or wealthier increased the risk of having all CVDRFs except for smoking. There is substantial loss of patients at each step of the care cascade for both diabetes and hypertension with less than 10% of the total

For peer review only - http://bmjopen.bmj.com/site/about/guidelines.xhtml

population with the conditions being screened, diagnosed, treated and controlled. The most common reasons for not seeking care were lack of knowledge and cost.

Conclusions In Sierra Leone CVDRFs are prevalent and access to care is low. Health system strengthening with a focus on increased access to quality care for CVDRFs is urgently needed.

For peer review only

For peer review only - http://bmjopen.bmj.com/site/about/guidelines.xhtml

INTRODUCTION

Non-communicable diseases (NCDs) such as cardiovascular disease and its risk factors are major health problems globally.[1] The reduction in deaths from infections including HIV has led to an aging population which has together with lifestyle transitions towards a high-calorie, low-activity and urban lifestyle, led to a high and rising prevalence of NCDs in lower and middle income countries (LMIC).[2-4] In fact, high blood pressure has become the largest contributor to premature mortality globally,[3,4] and cardiovascular diseases (including coronary heart disease and stroke) are the most common NCDs, globally responsible for an estimated 17.8 million deaths in 2017.[2] More than three quarters of these were in LMICs.[2]

However, surveillance of the prevalence of cardiovascular disease risk factors is very limited in the poorest countries in the world. Sierra Leone is a low-income country situated in West-Africa. It has a human development index of 0.419 (184 of 189 countries) and a maternal mortality ratio (1360 per 100 000 live births) and under-5 mortality rate (110.5 per 1000 live births) among the highest in the world.[5] The civil war from 1991-2002 disrupted infrastructure development, including that of the health system. Moreover, the 2013-2016 Ebola virus disease created a public health crisis and drew resources away from broader development of the health system.[6,7]

In recent years, both gross domestic product (GDP) and life expectancy at birth have increased in Sierra Leone.[8] In other countries (including those in sub-Saharan Africa) that have undergone a demographic transition, it has been accompanied by an increasing burden of cardiovascular disease risk factors (CVDRF) – such as diabetes and hypertension, dyslipidaemia, and overweight – with consequent macro- and microvascular disease outcomes – such as heart attacks, strokes, and blindness.[2,7] Unfortunately, although estimates of CVDRF prevalence from modelling studies exist, very little systematic, direct

For peer review only - http://bmjopen.bmj.com/site/about/guidelines.xhtml

measurement of the burden of CVDRF in the country has occurred; although small outdated studies have suggested a high burden of CVDRF.[9-13] These other studies are either more than 10 years old or have fewer than 700 participants. Additionally there is no information on whether and how sufferers are accessing care. Sierra Leone is developing its national policy and strategic plan for NCDs. To ensure efficient use of the already stretched healthcare resources, the strategic plan and its implementation needs to be informed by empirical information on the burden of risk factors and current access to care.[14] In order to provide evidence to assist health policy planning, this study aimed to describe the prevalence of CVDRF in people over 40 years old in Sierra Leone, access to care for those risk factors, and sociodemographic characteristics associated with CVDRF and access to care.

METHODS

Study setting

The study was conducted in the district of Bo, located in the Southern Province of Sierra Leone, and one of 16 districts in the country. It has well documented rural and urban areas and contains Sierra Leone's second largest city, Bo (appendix figure 1).[15] The demographics, socioeconomic circumstances, and geographical distribution of the population are similar to the larger Sierra-Leonean population.[15] In the last census in 2015 there were 575 478 inhabitants of Bo district with 66.1% (380 307) living in rural areas and 33.9% living in urban areas, mostly in Bo City. 17.4% (100 188) of the population are over 40 years of age.[15] Bo District has a mainly agriculture-based economy, but service-based industries are growing. Mende is the most used language, but Krio and English are also spoken.

For peer review only - http://bmjopen.bmj.com/site/about/guidelines.xhtml

Sampling strategy

A sample size of 1893 participants was targeted to allow detection of diabetes prevalence (the risk factor thought likely to have the lowest prevalence) of 4% with a precision of ±1%. To allow for non-response and non-availability of data, we oversampled by 20%. A sampling of individuals over 40 years of age was done from rural or urban areas in proportion with known patterns from the 2015 populations and housing census of habitation of these areas in the over 40s.[15] The 15 rural chiefdoms that comprise Bo District were listed in alphabetical order and 7 chiefdoms with separate geographic locations were chosen for the study using random number generator. Settlement groups or villages within these chiefdoms were identified and two were randomly chosen for study. Seven urban communities were randomly selected from 24 urban communities using similar methods of selection. Numbers of participants to sample from urban and rural areas were calculated based on the proportions of people living in these areas. In each urban community, numbers needed to study was 100. In each rural settlement or village, numbers needed to study was 93. If numbers were not achieved in the two selected areas, the next randomly ordered one was selected for study. Census information was not detailed enough to allow further identification of households with residents over 40 years old. Thus, data collection proceeded in each urban subdistrict or village, with data collectors starting at random points within each area and walking along a road or track sampling from every second household. Each household was permitted to enter no more than two people over 40 into the study. In villages where there were 93 households or fewer, all households were sampled. The geographical radius of the study was limited to 40 km from the centre of Bo to ensure accessibility. All chiefdoms and subdistricts in Bo were represented within this radius.

For peer review only

Data Collection

Data was collected electronically by trained staff using the ODK (Open Data Kit) platform.[16] from September-November 2018. The survey questionnaire was written in English but interviews were conducted in one of the local languages either Krio or Mende,

Survey questions asked about sociodemographic information - gender, age, highest level of education completed (no formal schooling, primary, junior secondary, senior secondary, higher education, or refused), employment the past 12 months (as government employee, non-government employee, self-employed, non-paid worker, student, homemaker, retired, unemployed able to work, unemployed unable to work, or refused), and marital status (as single, cohabiting, currently married, multiple partners, divorced, widowed, or refused). There were also 49 questions on household assets and construction materials. Questions on smoking, awareness of presence of CVDRF, and whether respondents were on treatment for these risk factors were based on the WHO STEPS survey; for those who reported suffering from a CVDRF, or had had a stroke, heart attack, or angina, whether care had been accessed, where care was accessed, and reasons for not accessing care were also asked.[11]

Height was measured using tape with participants standing with their backs, hips and heels against a wall and looking ahead horizontally (this method was validated using a Height Measure (SECA 213) during training). An Accuweight® digital body scale was used for measuring weight whilst wearing light clothing and without shoes.

Sitting blood pressure was measured using an Omron M6 AC LED Blood Pressure Monitor. Three measurements were taken with five minutes intervals between measurements. Blood samples were taken first thing in the morning after an 8 hour overnight fast. Glucose and cholesterol were measured using the Accutrend® Plus Blood Test Meter (Diagnostics Roche) point of care device.

For peer review only - http://bmjopen.bmj.com/site/about/guidelines.xhtml

Participant's fasting status was checked prior to the blood sample being taken, and those who reported not fasting were labelled as such. Cholesterol samples were obtained from every second participant, while glucose was measured from all participants. The conversion rate of 1.11 was used to convert capillary glucose to plasma glucose.[17]

Outcome measures

Body mass index (BMI) was defined as weight (measured in kilograms (kg)) divided by height (measured in meters squared) and classified as normal weight (<25kg/m^2) or overweight/obese (BMI ≥ 25kg/m^2). An additional analysis with normal and overweight (<30kg/m^2) versus obese (BMI ≥ 30kg/m^2) was also done. Diabetes was defined as fasting plasma glucose (FPG) ≥7.0 mmol/L (126 mg/dL), or as random plasma glucose (RPG) ≥11.1 mmol/L (200 mg/dL). Hypertension was defined as recorded systolic blood pressure ≥ 140 or diastolic ≥ 90mmHg, calculated using the average of the final two readings. Dyslipidaemia was defined as measured total cholesterol level ≥ 6.21 mmol/L, or low-density lipoprotein (LDL) ≥ 4.1 mmol/L, or high-density lipoprotein (HDL) < 1.19 mmol/L. Participants that reported they had taken drugs for diabetes, hypertension or dyslipidaemia within the last two weeks were classified as having these conditions irrespective of their biomarker measurements. Smoking was defined as: current smoker if participants either reported currently smoking or had ceased within in the last year, or non-smoking for others. Educational level was defined as having completed "any level of education" (primary, secondary or University) or "no completed education. Marital status was defined as married/cohabiting or single/widowed/divorced. Wealth quintiles were derived from the first principal component of household assets and construction materials using the method of Filmer and Pritchett.[18]

For peer review only - http://bmjopen.bmj.com/site/about/guidelines.xhtml

Access to healthcare

Self-reported access to care

Everyone with self-reported previous diagnosis of hypertension, diabetes, dyslipidaemia, angina, heart attack or stroke was asked if they had accessed care for their conditions in the last four weeks or three months. Reasons for not accessing care were explored for the ones who did not have self-reported access to care.

Construction of the care cascade

A cascade of care was constructed for diabetes and hypertension. The stages in the care cascade are:

1) Prevalent disease (the population defined as having hypertension or diabetes)

2) Ever been screened (the population who have had their blood pressure or glucose measured by a health personnel)

3) Prior diagnosis (the population who have ever been told by a doctor or other health care worker that they have hypertension or diabetes)

4) Currently on treatment (the population who have taken drugs for hypertension or diabetes in the last two weeks)

5) Disease control (the population who have their condition controlled to target at study measurement)

Entry into each subsequent stage of the cascade was contingent on an individual having achieved the previous stage. The population prevalence for diabetes and hypertension formed the denominators for all other stages of the respective care cascade. Additionally, the loss

For peer review only

For peer review only - http://bmjopen.bmj.com/site/about/guidelines.xhtml

from each step in the care cascade was calculated using the people who had achieved the previous step as the denominator.

Statistical analysis

Statistical analysis was done using SPSS v24 (IBM, New York). Descriptive statistics were described using mean and SD for normally distributed continuous variables and median and IQR for non-normally distributed variables. Univariate associations between independent variables (demographic characteristics) and outcomes (CVDRFs) were tested using Chi Squared tests and Kendalls Tau-B for categorical variables and Mann-Whitney and Spearman's Rho for continuous variables. Multivariable analyses were performed using binary logistic regression with forced entry of all independent variables. For hypertension, factors associated with achieving each step in the cascade were tested. This was not done for diabetes as numbers were too small for meaningful results. A sensitivity analysis using BMI>30 as a cut off was done (appendix table 1), and we decided to use age as categorical variable in the multivariable analysis due to non-linear association with some outcomes (for example demographic characteristics and CVDRF). Confidence intervals for proportions was calculated using according to a method described by Robert Newcombe derived from a procedure outlined by E.D Wilson.[19]

Probability weights for age and sex in Bo-South were calculated based upon the 2015 Population and Household Census.[15] All analyses were done using weight adjustments. Clustering at village level was adjusted for in the multivariable analyses.

For peer review only - http://bmjopen.bmj.com/site/about/guidelines.xhtml

Patient and public involvement statement

Participants were not directly involved in planning the study.

RESULTS

The final sample included 2071 individuals. The weighted demographic characteristics and prevalence of cardiovascular risk factors of the study population are presented in table 1. The unweighted proportions of demographic characteristics of participants with measured cholesterol versus not measured cholesterol are presented in appendix table 2. Those who had their cholesterol measured were similar to those who did not. However, there were fewer males who had cholesterol measured.

Population characteristics and risk factor prevalence

The population predominately lived in rural areas (62.9%) and 49.0% of the study population was female. The median age was 51.0 years, 67.4% had not completed any education and 72.6% were married/cohabiting. The prevalence of hypertension was 49.6% (95% CI 49.3-50.0) whilst the prevalence of diabetes and dyslipidaemia were 3.5% (95% CI, 3.4-3.6) and 6.7% (95% CI, 6.5-7.0) respectively. Overweight or obesity (BMI $\geq$ 25kg/m^2) was present in 26.5% (95% CI, 26.3-26.8) of the study population and 25.6% (95% CI 25.4-25.9) of the participants were current or recent (within the last year) smokers. Altogether, 77.1% (95% CI, 76.6-77.5) of the study population had at least one CVDRF when including cholesterol (and limiting the denominator to those 789 who had cholesterol measured), whilst when excluding cholesterol as a variable (and with a denominator of 1896 who had information on all other CVDRF) the prevalence of at least one CVDRF was 74.5%. (95 CI, 74.3-74.8).

For peer review only - http://bmjopen.bmj.com/site/about/guidelines.xhtml

For peer review only

Univariate associations between demographic characteristics and CVDRF are presented in appendix table 3.

In the multivariable analysis (table 2) living in an urban area was independently associated with all CVDRFs except for dyslipidaemia (which was more prevalent in those living in rural areas). Male sex was independently associated with lower prevalence of CVDRF with the exceptions of smoking and the presence of any risk factor. Increasing age was independently associated with increasing prevalence of hypertension, diabetes or dyslipidaemia, and with a decreased prevalence of being overweight or smoking. The prevalence of CVDRFs according to age group and sex is shown in figure 1. Having any education compared to no complete education was independently associated with increased prevalence of all CVDRFs expect for smoking. Being married or cohabiting was independently associated with lower prevalence of all CVDRFs except for diabetes and obesity. Wealth remained independently associated with all CVDRFs except for smoking, where increasing wealth quintile was associated with a lower prevalence of smoking.

Access to healthcare

A total of 496 participants reported a previous diagnosis of hypertension, diabetes or dyslipidaemia, angina, heart attack or stroke. Of these, only 88 (17.74%) stated that they had accessed health care for their cardiovascular diseases in the last three months and only 8.87% had accessed health care in the last four weeks. The most common reasons for not accessing healthcare were thinking that it wasn't necessary (47.0%) or that it was too expensive (24.5%). Everyone who accessed care in the last three months visited a modern health

For peer review only - http://bmjopen.bmj.com/site/about/guidelines.xhtml

facility, with 35.5% visiting community-based health service, and 63.2% a hospital-based health service. Nobody reported having visited a traditional healer for their condition.

The cascade of care for hypertension is shown in figure 2. Among those with hypertension, 59.2% reported that they had their blood pressure measured by a health care professional (screened), and 33.2% had ever been diagnosed with hypertension. There was a substantial loss to care at both steps, 40.8% and 44.0% respectively. Only 14.7% of people with hypertension were currently on treatment (taken medication for hypertension in the last two weeks), and of the people who were currently on treatment 31.2% achieved control. The last step of the cascade, being controlled, had the biggest loss to care from the previous step of 68.8%. In the multivariable analysis of the hypertension cascade, (table 3) people living in an urban area were significantly more likely to pass through all the steps of the cascade apart from being diagnosed. Women were more likely than men to be screened or diagnosed, but not treated; men were more likely than women to be controlled. There was no clear relationship between age groups and progress through the cascade. Having some education or being wealthier were significantly associated with passing through the first three steps of the cascade, but not with being controlled.

The cascade of care for diabetes is presented in figure 3. Out of all the people with diabetes in our study population (hyperglycaemic on measurement or taken medication in the last two weeks), the largest loss to care was at the stage of screening with only 57% of participants reporting that they had had their blood sugar measured at any time previously. There was a more modest loss to care for the next step with 32.9% of the participants with diabetes reporting that they had ever been told that they have diabetes. For the next step only 19% of the participants with diabetes reported that they had been taking treatment for diabetes in the

For peer review only

last two weeks. Finally, 8.6% of the total population with diabetes had achieved control of their disease which is less than half the population that reported that they were on treatment. For diabetes the sample size was too small to do multivariable analysis with demographic characteristics in the different steps in the cascade.

DISCUSSION

This paper reports one of the first studies to provide estimates of the prevalence of all CVDRFs in Sierra Leone; it is the first that we are aware of to publish on access to care for CVDRFs. Our data suggest that the prevalence of CVDRFs in Sierra Leone is high with 75% of the population over 40 having at least one CVDRF. The risk of having a CVDRF increased with age, and CVDRFs was more common in the urban population, among women, unmarried people and individuals with education and in the highest wealth quintile. Smoking was very common among men, giving them a higher overall risk of having at least one CVDRF. Also, our analysis revealed that there are very high rates of unmet need for hypertension and diabetes care. Less than 20% of the population with hypertension, diabetes and dyslipidaemia accessed health care in the last three months.

Although we sampled only one area in Sierra Leone, the population structure is similar to other areas in Sierra Leone except for Freetown.[15] Thus our findings give insight into the likely prevalence and associations across the country. Indeed, our estimate of hypertension of about 50% is similar to that found previously in Sierra Leone in the same age group in other areas.[9,10] There are very little data available on diabetes from Sierra Leone, but the most recent estimates, both empirical and modelled, were much higher than we found in our study.[12,20] For example the NCD Risk collaboration estimated prevalence of diabetes to be

For peer review only - http://bmjopen.bmj.com/site/about/guidelines.xhtml

7.1% (95% CI 3.5-12.1) in 2014.[20] The prevalence of diabetes in urban areas in our material (5.5%) was however similar to a previous study (6.2%) from 2012-2014 collected in only urban areas of Bo.[17] An older study conducted in Bo in 1997 reported a lower prevalence of 2.4% in the urban population and 0% in the rural population.[21] Diabetes prevalence might be rising with time, but the methodologies used in the previous studies makes comparisons difficult. Both the previous studies were also much smaller in sample size (n=694 and n=501) than ours, and likely underpowered.

In contrast, the prevalence of hypertension in our study is higher than previous empirical data from the WHO STEPS survey conducted in 2009, and which found hypertension in 37% of males and 33% of females.[11] The population sampled in the previous WHO STEPS survey was younger (25-65 years) than in our study though, and the prevalence of hypertension is also likely to have increased in the past years.

Other areas in West-Africa have also reported a similarly high prevalence of CVDRFs to what we have found, although prevalence of hypertension in Sierra Leone in our study is higher than other regional estimates from countries like Nigeria and Ghana.[3 22-25] 25% of the population in our sample were overweight or obese which is surprising for one of the poorest countries in the world. However, our estimates of overweight/obesity are slightly lower than those derived from the WHO STEPS survey from 2009,[11] and lower than those reported from Nigeria, so it is unlikely that our findings over-estimate the prevalence.[24] The geographical, and socioeconomic and education balance of most CVDRF that we found are also reflective of findings from other studies in the region.[24,25] However, in other studies, CVDRFs like diabetes and hypertension are more prevalent in males in contrast to our findings.[24,25] Still, overall, males actually have a higher risk of having at least one CVDRF than females in our sample. This makes males a vulnerable group when it comes CVDRFs, especially since the

For peer review only - http://bmjopen.bmj.com/site/about/guidelines.xhtml

cascade analysis suggests that they are less likely to enter into the healthcare system for their conditions than women.

The low prevalence of people with hypertension being controlled for their condition is similar to what has been previously shown in countries in Sub-Saharan Africa.[4] Regarding diabetes, other studies have shown that many low-income countries in sub-Saharan Africa perform better than Sierra Leone on access to care with an average of more than 15-20% of the patients achieving control of the disease.[26,27] However, similar to our findings, the biggest loss to care was at the stage of screening.[4] Although there are no studies done on the access to care for CVDRFs in Sierra Leone, previous studies on HIV-care has shown that the loss to care is substantial with only 22.8% of patients with newly diagnosed HIV receiving effective treatment.[28] It might be tenuous to compare HIV care and care for CVDRF, as HIV care receives substantial financial support from donors. Care for HIV is also largely separated from the public health care system, and health seeking behaviour for HIV is affected by stigma. Nevertheless, it is another indication that the health system in Sierra Leone finds it challenging to provide long-term follow up care for patients with chronic disorders.

Living in an urban area was a strong predictive factor for passing through the cascade steps and achieving control of hypertension. Women were more likely to be screened and diagnosed for hypertension than men which could be due to women accessing maternal and child health care (which has been a focus of healthcare efforts in Sierra Leone), gender norms, and facility opening hours. It is important to ensure that efforts are made to encourage and retain men in care. People with higher education and in the highest wealth quintile were also more likely to access care; similar to previous findings regarding access to hypertension care in LMICs.[4,29] Poorer and uneducated people are also more likely to experience

For peer review only

catastrophic health expenditure on accessing care for non-communicable diseases, and investments in improving hypertension care present an opportunity to reduce health inequalities between socioeconomic groups. Even if health care is free, which in Sierra Leone is the case for the 'destitute', Ebola survivors, pregnant women, lactating women, or children under 5,[30] accessing care still require transport costs and is time lost from income generating activity.[31] That we found that the most common reasons for not accessing care included cost suggests that addressing this barrier is key to providing care for sufferers of CVDRF in Sierra Leone. Interestingly, the people most likely to access care in our study (high education and wealth) were less likely to succeed at the last step in the cascade by achieving control of their condition. One reason for this could be that medications are not taken regularly. However, this finding could also be due to lack of study power due to the low number of people reaching the last step in the cascade.

This study is one of the first studies to report prevalence of multiple CVDRFs in such a large sample from Sierra Leone and the first study to report access to care for these. The study sample is larger than any previous studies on CVDRF in Sierra Leone, and the data sampling and analysis were done in a rigorous way to avoid potential biases. Bo also consists of urban and rural areas that are similar to rest of Sierra Leone.[15] Hence the sample should be comparable to the rest of the population.

There are several limitations in this study. First of all, we could not measure cholesterol in the total population due to lack of resources. However, appendix table 2 shows that there were few differences between the populations with measured cholesterol versus those without cholesterol measurements. The data collection was also limited to within 40 km of Bo City due to accessibility from Bo and travel times. However, all chiefdoms were represented

For peer review only - http://bmjopen.bmj.com/site/about/guidelines.xhtml

within this distance and were entered into the randomisation. It is unlikely that those areas further from Bo, as an urban centre, would be different to those not selected, as areas more than 40 km from Bo were close to other conurbations in neighbouring districts. We did not control for clustering at household level as few houses supplied more than one participant.

In this study we have showed that the prevalence of CVDRFs in one of poorest populations in the world is remarkably high, and the access to care is low. This should have major implications for health policy and planning in Sierra Leone in the years to come. Early deaths and disability due to cardiovascular disease can disrupt the little economic development the country has experienced in recent years and should be given more attention. There is an urgent need to plan where appropriate interventions can be implemented in the most efficient way to make the most of the country's limited health care resources, in order to prevent CVDRFs and its consequences.

CONCLUSIONS

This study shows that about 75% of the population in Bo, Sierra Leone, has at least one cardiovascular risk factor and access to care is very low. In particular, men living in rural areas have a high cardiovascular risk profile and do not access care. The results from this study can inform national plans for cardiovascular disease prevention and management.

Contributors JD, MDW, RN and IB conceived and designed the overall study. JD, TB, HW, RA and JL coordinated baseline data collection and preparation. JD, MDW, RN and IB contributed to the design of the household survey. MLO conducted the analysis, and wrote, and revised the manuscript. JD supervised the analysis, write up, and development of the manuscript. All authors substantively reviewed manuscripts, inputted into revisions, and approved the final manuscript.

Funding Support for the study was given by the Wellcome Trust.

Competing interest The authors report no competing interest in conducting this study.

Patient consent for publication Not required.

For peer review only - http://bmjopen.bmj.com/site/about/guidelines.xhtml

Ethical approval Ethical approval was sought and given from the Sierra Leone Ethical and Scientific Review Committee and the BDM Research Ethics sub-committee at King's College London (HR-17/18-7298). Consent to undertake the study was obtained from each village chief or community leader. Consent was obtained from all individuals participating in the study. In the event were participants were illiterate, the consent form was read out to them in the local language and an inked-thumb signature obtained. Information was fed back to patients if they had abnormal measurements and they were referred to a local health care facility.

Data availability statement Data are not publicly available as consent was not given by participants for this to take place.

Acknowledgements
MDW acknowledges support from the NIHR Newcastle Biomedical Research Centre.
We thank the data collectors (DC) and field manager (FM) who worked on this study for their tireless commitment. These include: Ramatu Senesie DC; Allieu Abu Sheriff DC; Albert Sidikie Sama FM; Abdulai Kamara DC; Umu Binta Bah DC; Michael Dawson DC; Christiana Pratt DC; Michael E. Garrick DC; Peter Tamba Morsay DC; Francess Koker DC; Ismael Vandi DC; Samuel Kamanda DC; Wilfred A. U. Jimmy DC-Team Supervisor; Yvonne Vincent DC; Abu Bakarr Mansaray DC; Mariama Jalloh DC- Team Supervisor; In addition, we also want to thank and acknowledge the interns (Kadijatu Assiatu Kargbo; Amara Vandi Fomba; Rita kallon; Veronica Manty Marrah; Carpenter Emmanuel; Bangura A. Ronald; Kpallu Kpakila Sahr; Habibatu Adama Konuwa who supported our research team other research activities.

References

1 Roth GA, Forouzanfar MH, Moran AE, et al. Demographic and epidemiologic drivers of global cardiovascular mortality. *N Engl J Med* 2015; 372(14): 1333-41.

2 GBD 2017 Causes of Death Collaborators. Global, regional, and national age-sex-specific mortality for 282 causes of death in 195 countries and territories, 1980-2017: a systematic analysis for the Global Burden of Disease Study 2017. *Lancet* 2018; 392(10159): 1736-88.

3 NCD Risk Factor Collaboration (NCD-RisC) Worldwide trends in blood pressure from 1975 to 2015: a pooled analysis of 1479 population-based measurement studies with 19.1 million participants. *Lancet* 2017; 389(10064): 37-55.

4 Geldsetzer P, Manne-Goehler J, Marcus ME, et al. The state of hypertension care in 44 low-income and middle-income countries: a cross-sectional study of nationally representative individual-level data from 1.1 million adults. *Lancet* 2019; 394(10199): 652-62.

5 The World Bank. Sierra Leone. 2019. https://data.worldbank.org.

6 World Health Organisation. Ebola situation report 30 March. 1th edn. Geneva. 2016. https://www.who.int/csr/disease/ebola/situation-reports/archive/en/.

7 Samba T, Bhat P, Owiti P, et al. Non-communicable diseases in the Western Area District, Sierra Leone, before and during the Ebola outbreak. *Public Health Action* 2017; 7(Suppl 1): S16-S21.

8 UNDP. Human Development Indices and Indicators: 2018 Statistical Update. 2018. http://hdr.undp.org/sites/all/themes/hdr_theme/country-notes/SLE.pdf.

9 Awad M, Ruzza A, Mirocha J, et al. Prevalence of hypertension in the Gambia and Sierra Leone, western Africa: a cross-sectional study. *Cardiovasc J Afr* 2014; 25(6): 269-78.

10 Meehan KA, Bankoski AJ, Tejan E, et al. Hypertension in Bo, Sierra Leone. *Ethn Dis* 2011; 21(2): 237-42.

11 World Health Organisation. WHO STEPs Sierra Leone. 2009. https://www.who.int/ncds/surveillance/steps/2009_Sierra_Leone_FactSheet_EN.pdf.

12 Sundufu AJ, Bockarie CN, Jacobsen KH. The prevalence of type 2 diabetes in urban Bo, Sierra Leone, and in the 16 countries of the West Africa region. *Diabetes Metab Res Rev* 2017; 33(7).

For peer review only - http://bmjopen.bmj.com/site/about/guidelines.xhtml

13 Lisk DR, Williams DE, Slattery J. Blood pressure and hypertension in rural and urban Sierra Leoneans. *Ethn Dis* 1999; 9(2): 254-63.

14 World Health Organisation. National capacity to address and respond to NCDs: Existence of operational policies, strategies, or action plans. 2016. http://www.who.int/gho/ncd/health_system_response/policy_text/en/.

15 Statistics Sierra Leone. 2015 Population and Housing Census. 2015. https://www.statistics.sl/images/StatisticsSL/Documents/final-results_-2015_population_and_housing_census.pdf.

16 Hartung C. Open Data Kit: Tools to Build Information Services for Developing Regions. 2010.

17 D'Orazio P, Burnett RW, Fogh-Andersen N, et al. Approved IFCC recommendation on reporting results for blood glucose: International Federation of Clinical Chemistry and Laboratory Medicine Scientific Division, Working Group on Selective Electrodes and Point-of-Care Testing (IFCC-SD-WG-SEPOCT). *Clin Chem Lab Med* 2006; 44(12): 1486-90.

18 Filmer D, Pritchett LH. Estimating wealth effects without expenditure data--or tears: an application to educational enrollments in states of India. *Demography* 2001; 38(1): 115-32.

19 Newcombe RG. Two-sided confidence intervals for the single proportion: comparison of seven methods. *Stat Med* 1998; 17(8): 857-72.

20 NCD Risk Factor Collaboration (NCD-RisC). Worldwide trends in diabetes since 1980: a pooled analysis of 751 population-based studies with 4.4 million participants. *Lancet* 2016; 387(10027): 1513-30.

21 Ceesay MM, Morgan MW, Kamanda MO, Willoughby VR, Lisk DR. Prevalence of diabetes in rural and urban populations in southern Sierra Leone: a preliminary survey. *Trop Med Int Health* 1997; 2(3): 272-7.

22 Cappuccio FP, Micah FB, Emmett L, et al. Prevalence, detection, management, and control of hypertension in Ashanti, West Africa. *Hypertension* 2004; 43(5): 1017-22.

23 Ezejimofor M, Uthman O, Chen YF, et al. Magnitude and pattern of hypertension in the Niger Delta: a systematic review and meta-analysis of community-based studies. *J Glob Health* 2018; 8(1): 010420.

24 Oguoma VM, Nwose EU, Skinner TC, Digban KA, Onyia IC, Richards RS. Prevalence of cardiovascular disease risk factors among a Nigerian adult population: relationship with income level and accessibility to CVD risks screening. *BMC Public Health* 2015; 15: 397.

25 Kodaman N, Aldrich MC, Sobota R, et al. Cardiovascular Disease Risk Factors in Ghana during the Rural-to-Urban Transition: A Cross-Sectional Study. *PLoS One* 2016; 11(10): e0162753.

26 Manne-Goehler J, Geldsetzer P, Agoudavi K, et al. Health system performance for people with diabetes in 28 low- and middle-income countries: A cross-sectional study of nationally representative surveys. *PLoS Med* 2019; 16(3): e1002751.

27 Price AJ, Crampin AC, Amberbir A, et al. Prevalence of obesity, hypertension, and diabetes, and cascade of care in sub-Saharan Africa: a cross-sectional, population-based study in rural and urban Malawi. *Lancet Diabetes Endocrinol* 2018; 6(3): 208-22.

28 Kelly JD, Schlough GW, Conteh S, Barrie MB, Kargbo B, Giordano TP. The Majority of the Pre-Antiretroviral Population Who Were Lost to Follow-Up Stopped Their Care in Freetown, Sierra Leone: A 12-Month Prospective Cohort Study Starting with HIV Diagnosis. *PLoS One* 2016; 11(2): e0149584.

29 Jan S, Laba TL, Essue BM, et al. Action to address the household economic burden of non-communicable diseases. *Lancet* 2018; 391(10134): 2047-58.

30 Treacy L, Bolkan HA, Sagbakken M. Distance, accessibility and costs. Decision-making during childbirth in rural Sierra Leone: A qualitative study. *PLoS One* 2018; 13(2): e0188280.

31 Chimbindi N, Bor J, Newell ML, et al. Time and Money: The True Costs of Health Care Utilization for Patients Receiving "Free" HIV/Tuberculosis Care and Treatment in Rural KwaZulu-Natal. *J Acquir Immune Defic Syndr* 2015; 70(2): e52-60.

For peer review only

For peer review only - http://bmjopen.bmj.com/site/about/guidelines.xhtml

Table 1 Weighted demographic characteristics and prevalence of cardiovascular risk factors in Bo, Sierra Leone (n=2071)

parameter	group	% using weights
Place of living	Rural	62.90
	Urban	37.10
Gender	Female	49.00
	Male	51.00
Age median (IQR) n = 2062	Years	51.0 (45.0-63.0)
education level n = 2070	No completed education	67.40
	Any education	32.60
marital status n = 2069	Married/Cohabiting	72.60
	Single/widowed/divorced	27.40
wealth quintile n = 1991	1	20.50
	2	20.50
	3	20.00
	4	19.90
	5	19.10
Cardiovascular disease risck factors (CVDRF)	Hypertension n =2070	49.60
	Mean (SD) SBP	136.19 (25.24)
	Mean (SD) DBP	87.52 (14.11)
	Diabetes n = 2019	3.50
	Dyslipidaemia n = 840	6.70
	Overweight/obesity n = 1947	26.50
	Smoking	25.60
	One CVD risk factor or more out of a possible 7 - including cholesterol (n = 789)	Including cholesterol 77.10
	One CVD risk factor or more out of a possible 6 - excluding cholesterol (n = 1896)	Excluding cholesterol 74.50

For peer review only

Table 2 Multivariable associations between demographic characteristics and cardiovascular risk factors including cholesterol (n=2071)

parameter	group	Hypertension		Diabetes		Dyslipidaemia		Overweight/obese		Smoking		Total CVD risk factors incl. chol		Total CVD risk factors exl. chol	
		OR (95% CI)	P-value	OR (95% CI)	P-value	OR (95% CI)	P-value	OR (95% CI)	P-value	OR (95% CI)	P-value	OR (95% CI)	P-value	OR (95% CI)	P-value
Place of living	Rural	Referent	-	Referent	-	Referent	-	Referent	-	Referent	-	Referent	-	Referent	-
	Urban	1.04 (1.01-1.08)	0.014	1.46 (1.34-1.60)	<0.001	0.84 (0.75-0.93)	0.001	1.17 (1.12-1.21)	<0.001	1.13 (1.08-1.17)	<0.001	0.99 (0.93-1.05)	0.614	1.06 (1.02-1.10)	0.002
Gender	Female	Referent	-	Referent	-	Referent	-	Referent	-	Referent	-	Referent	-	Referent	-
	Male	0.78 (0.75-0.80)	<0.001	0.75 (0.69-0.82)	<0.001	0.88 (0.80-0.97)	0.013	0.31 (0.30-0.32)	<0.001	9.15 (8.76-9.54)	<0.001	1.6 (1.52-1.70)	<0.001	1.43 (1.38-1.48)	<0.001
Age	40-49	Referent	-	Referent	-	Referent	-	Referent	-	Referent	-	Referent	-	Referent	-
	50-59	1.75 (1.69-1.81)	<0.001	2.10 (1.91-2.32)	<0.001	1.38 (1.25-1.53)	<0.001	0.84 (0.81-0.87)	<0.001	0.84 (0.81-0.88.0)	<0.001	0.93 (0.88-0.99)	0.023	1.15 (1.11-1.20)	<0.001
	60-69	2.35 (2.26-2.45)	<0.001	2.77 (2.50-3.07)	<0.001	1.36 (1.22-1.53)	<0.001	0.85 (0.81-0.89)	<0.001	0.58 (0.56-0.61)	<0.001	1.25 (1.16-1.35)	<0.001	1.70 (1.60-1.81)	<0.001
	70-79	3.43 (3.27-3.61)	<0.001	3.46 (3.07-3.89)	<0.001	1.76 (1.52-2.05)	<0.001	0.70 (0.66-0.75)	<0.001	0.36 (0.33-0.38)	<0.001	2.24 (2.00-2.51)	<0.001	1.25 (1.16-1.34)	<0.001
	>80	3.13 (2.96-3.32)	<0.001	1.76 (1.69-1.99)	<0.001	0.98 (0.81-1.19)	0.835	0.49 (0.45-0.53)	<0.001	0.52 (0.48-0.56)	<0.001	1.22 (1.09-1.38)	0.001	1.07 (1.16-1.34)	<0.001
Education level	No complete education	Referent	-	Referent	-	Referent	-	Referent	-	Referent		Referent		Referent	-
	Any education	1.17 (1.14-1.21)	<0.001	1.83 (1.69-1.99)	<0.001	1.08 (0.98-1.18)	0.111	1.63 (1.57-1.69)	<0.001	0.86 (0.83-0.89)	<0.001	0.91 (0.86-0.96)	0.001	1.07 (1.04-1.11)	<0.001
Marital status	Single/divorced/widow	Referent	-	Referent	-	Referent	-	Referent	-	Referent	-	Referent	-	Referent	-
	Married/Cohabiting	0.8 (0.78-0.83)	<0.001	1.01 (0.93-1.11)	0.785	0.62 (0.56-0.68)	<0.001	1.1 (1.06-1.15)	<0.001	0.8 (0.77-0.84)	<0.001	0.81 (0.76-0.86)	<0.001	0.84 (0.81-0.88)	<0.001

For peer review only - http://bmjopen.bmj.com/site/about/guidelines.xhtml

Wealth quintile	1	Referent	-	Referent	-	Referent	-	Referent	-	Referent	-	Referent	-	Referent	-
	2	0.83 (0.79-0.86)	<0.001	1.31 (1.14-1.52)	<0.001	1.84 (1.47-2.29)	<0.001	1.42 (1.34-1.50)	<0.001	0.96 (0.92-1.01)	0.105	1.06 (0.98-1.14)	0.142	1.1 (1.05-1.15)	<0.001
	3	0.99 (0.95-1.03)	0.698	1.2 (1.04-1.39)	0.014	2.36 (1.90-2.29)	<0.001	1.66 (1.57-1.76)	<0.001	0.71 (0.68-0.75)	<0.001	0.87 (0.81-0.94)	0.001	0.91 (0.87-0.95)	0.001
	4	1.3 (1.25-1.36)	<0.001	1.64 (1.42-1.88)	<0.001	6.19 (5.07-7.56)	<0.001	3 (2.84-3.16)	<0.001	0.51 (0.49-0.54)	<0.001	1.41 (1.30-1.53)	<0.001	1.32 (1.25-1.38)	<0.001
	5	1.6 (1.52-1.69)	<0.001	2.7 (2.34-3.12)	<0.001	11.16 (9.05-13.76)	<0.001	5.11 (4.81-5.44)	<0.001	0.39 (0.37-0.42)	<0.001	2.46 (2.23-2.73)	<0.001	1.62 (1.53-1.72)	<0.001

For peer review only

For peer review only - http://bmjopen.bmj.com/site/about/guidelines.xhtml

Table 3 Multivariate associations between demographic characteristics and access to care for hypertension for people with hypertension (n=1092)

parameter	group	Screened (n=1092)		Diagnosis (n=646)		Treated (n=362)		Controlled (n=160)	
		OR (95% CI)	P-value	OR (95% CI)	P-value	OR (95% CI)	P-value	OR (95% CI)	P-value
Place of living	Rural	Referent	-	Referent	-	Referent	-	Referent	-
	Urban	1.61 (1.53-1.68)	<0.001	0.97 (0.91-1.03)	0.325	1.36 (1.23-1.50)	<0.001	2.13 (1.77-2.58)	<0.001
Gender	Female	Referent	-	Referent	-	Referent	-	Referent	-
	Male	0.70 (0.67-0.73)	<0.001	0.79 (0.74-0.84)	<0.001	1.13 (1.02-1.25)	0.015	1.12 (0.97-1.30)	0.121
Age	40-49	Referent	-	Referent	-	Referent	-	Referent	-
	50-59	0.91 (0.87-0.96)	0.001	1.36 (1.28-1.45)	<0.001	1.64 (1.49-1.80)	<0.001	0.95 (0.82-1.11)	0.535
	60-69	1.49 (1.41-1.58)	<0.001	1.01 (0.94-1.07)	0.874	2.15 (1.93-2.39)	<0.001	0.89 (0.76-1.04)	0.141
	70-79	1.22 (1.14-1.30)	<0.001	0.55 (0.51-0.60)	<0.001	1.44 (1.25-1.65)	<0.001	1.13 (0.93-1.38)	0.221
	>80	0.63 (0.59-0.68)	<0.001	0.72 (0.64-0.80)	<0.001	1.55 (1.29-1.87)	<0.001	1.09 (0.84-1.42)	0.516
Education level	No complete education	Referent	-	Referent	-	Referent	-	Referent	-
	Any education	1.78 (1.69-1.86)	<0.001	1.09 (1.03-1.16)	0.002	2.93 (2.69-3.18)	<0.001	0.70 (0.62-0.80)	<0.001
Marital status	Single/divorced/widow	Referent	-	Referent	-	Referent	-	Referent	-
	Married/Cohabiting	1.02 (0.97-1.07)	0.465	0.95 (0.89-1.01)	0.12	0.67 (0.61-0.73)	<0.001	1.17 (1.02-1.33)	0.022
Wealth quintile	1	Referent	-	Referent	-	Referent	-	Referent	-
	2	1.22 (1.15-1.30)	<0.001	1.59 (1.45-1.75)	<0.001	2.40 (1.97-2.91)	<0.001	0.25 (0.18-0.37)	<0.001
	3	1.77 (1.67-1.88)	<0.001	1.63 (1.50-1.79)	<0.001	2.95 (2.47-3.53)	<0.001	0.17 (0.12-0.22)	<0.001

For peer review only - http://bmjopen.bmj.com/site/about/guidelines.xhtml

	4	2.63 (2.47-2.80)	<0.001	1.45 (1.33-1.58)	<0.001	5.37 (4.52-6.39)	<0.001	0.16 (0.12-0.22)	<0.001
	5	4.21 (3.91-4.54)	<0.001	2.24 (2.04-2.46)	<0.001	4.97 (4.16-5.93)	<0.001	0.28 (0.20-0.39)	<0.001

For peer review only

Legends figure 1 Prevalence of cardiovascular risk factors according to age and sex

Legends figure 2 Cascade of care for hypertension using % of the population with the disease who had previously been tested ("screened"), knew of their condition ("diagnosed), were on treatment ("treated"), or were controlled to target ("controlled"). The loss to care at each step is described by the black arrows.

Legends figure 3 Cascade of care for diabetes using % of the population with the disease who had previously been tested ("screened"), knew of their condition ("diagnosed), were on treatment ("treated"), or were controlled to target ("controlled"). The loss to care at each step is described by the black arrows.

For peer review only

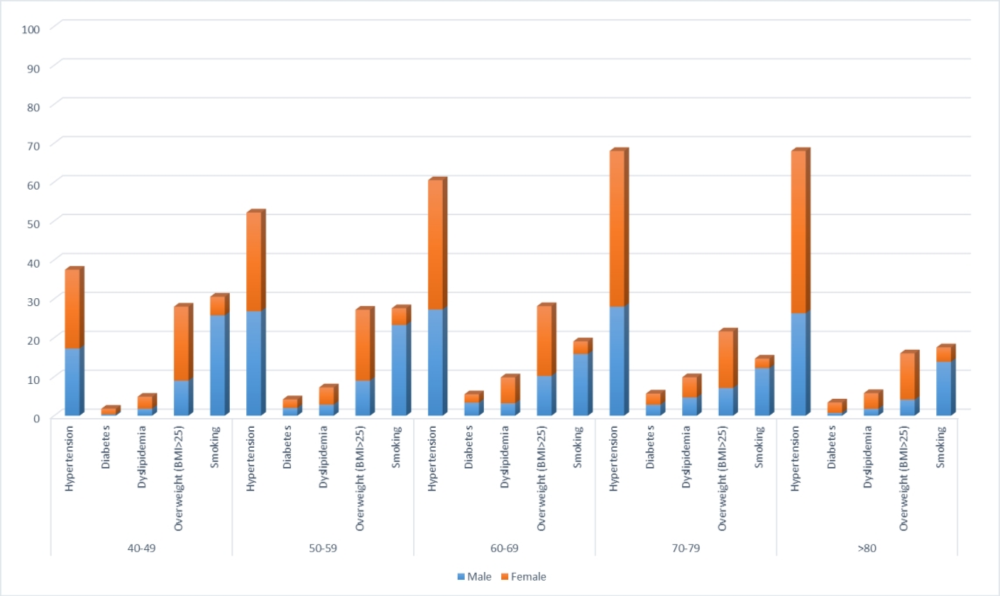

Figure 1 Prevalence of cardiovascular risk factors according to age and sex

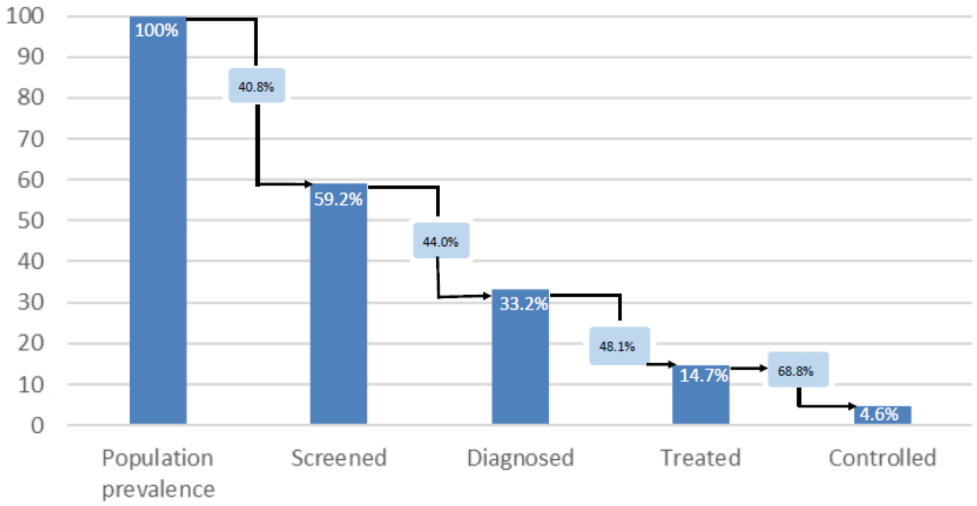

Figure 2 Cascade of care for hypertension using % of the population with the disease who had previously been tested ("screened"), knew of their condition ("diagnosed), were on treatment ("treated"), or were controlled to target ("controlled"). The loss to care at each step is described by the black arrows.

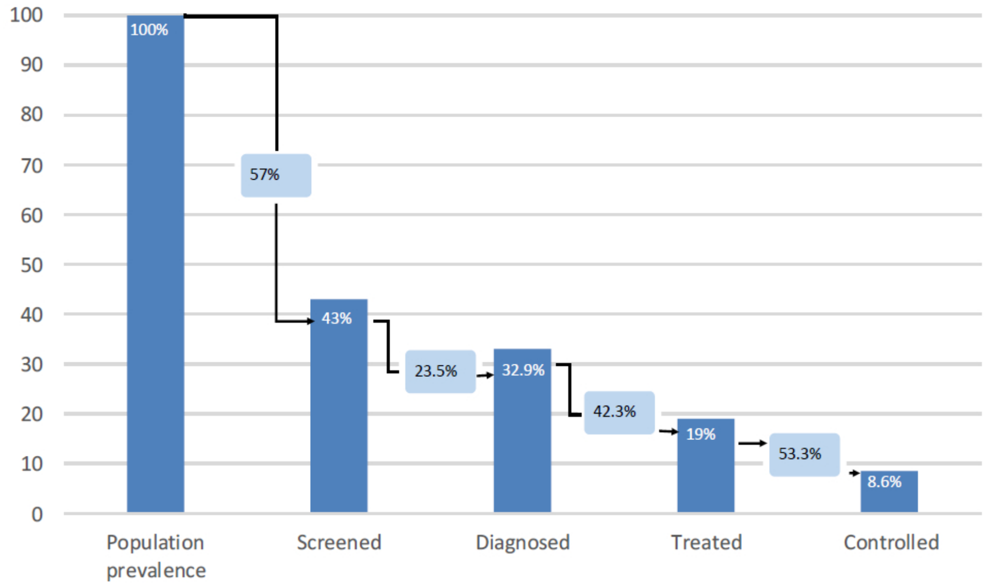

Figure 3 Cascade of care for diabetes using % of the population with the disease who had previously been tested ("screened"), knew of their condition ("diagnosed), were on treatment ("treated"), or were controlled to target ("controlled"). The loss to care at each step is described by the black arrows.

Appendix figure 1 Map of Bo Districts, Sierra Leone

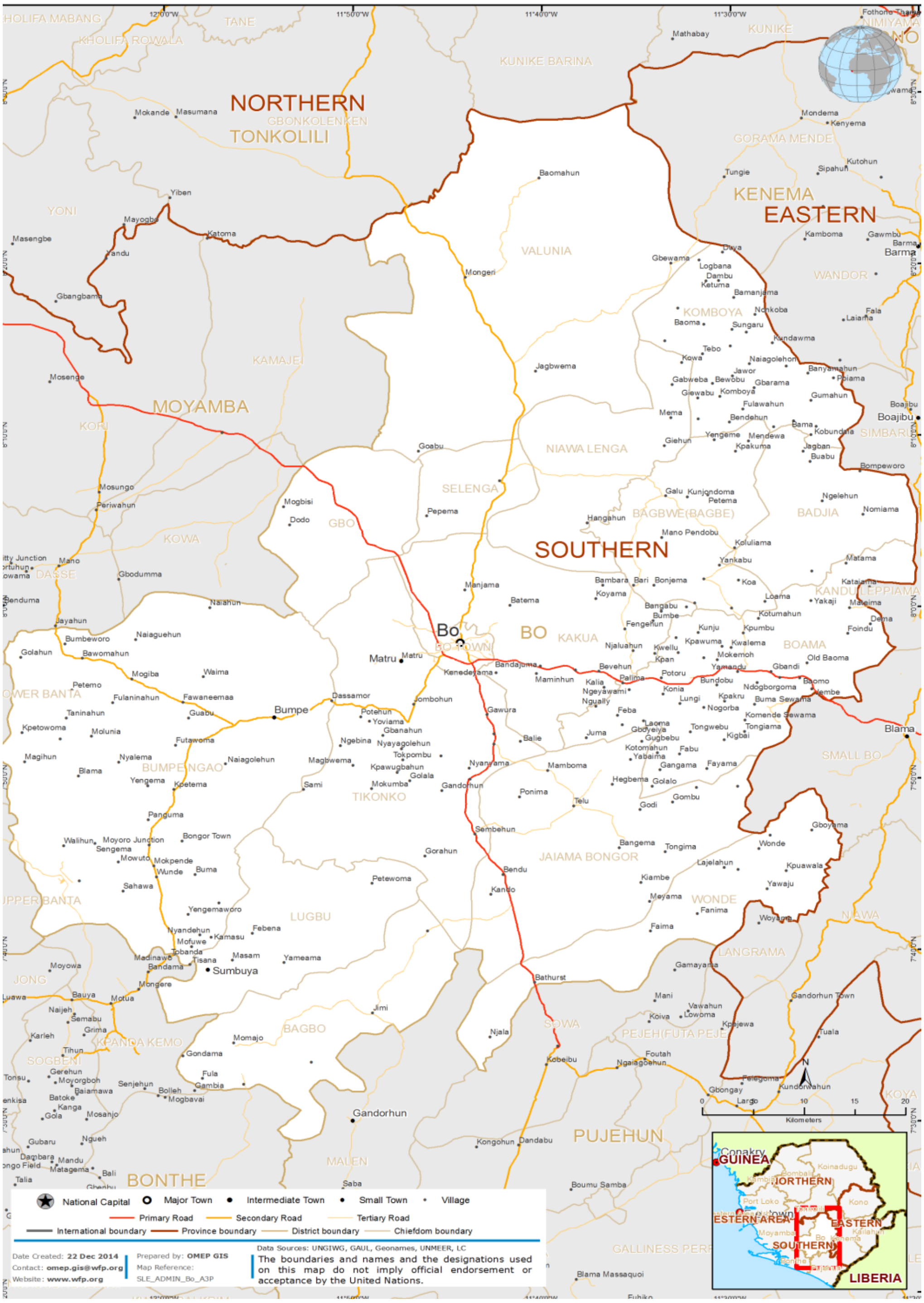

Appendix table 1 Sensitivity analysis with BMI >30 of multivariable associations between demographic characteristics and cardiovascular risk (n=2071)

parameter	group	Hypertension		Diabetes		Dyslipidaemia		Obesity (BMI>30)		Smoking		Total CVD risk factors incl. chol		Total CVD risk factors exl. chol	
		OR (95% CI)	P-value	OR (95% CI)	P-value	OR (95% CI)	P-value	OR (95% CI)	P-value	OR (95% CI)	P-value	OR (95% CI)	P-value	OR (95% CI)	P-value
Place of living	Rural	Referent	-	Referent	-	Referent	-	Referent	-	Referent	-	Referent	-	Referent	-
	Urban	1.04 (1.01-1.08)	0.014	1.46 (1.34-1.60)	<0.001	0.84 (0.75-0.93)	0.001	1.4 (1.33-1.48)	<0.001	1.13 (1.08-1.17)	<0.001	0.99 (0.93-1.05)	0.614	1.06 (1.02-1.10)	0.002
Gender	Female	Referent	-	Referent	-	Referent	-	Referent	-	Referent	-	Referent	-	Referent	-
	Male	0.78 (0.75-0.80)	<0.001	0.75 (0.69-0.82)	<0.001	0.88 (0.80-0.97)	0.013	0.24 (0.23-0.26)	<0.001	9.15 (8.76-9.54)	<0.001	1.6 (1.52-1.70)	<0.001	1.43 (1.38-1.48)	<0.001
Age	40-49	Referent	-	Referent	-	Referent	-	Referent	-	Referent	-	Referent	-	Referent	-
	50-59	1.75 (1.69-1.81)	<0.001	2.10 (1.91-2.32)	<0.001	1.38 (1.25-1.53)	<0.001	0.74 (0.70-0.79)	<0.001	0.84 (0.81-0.880	<0.001	0.93 (0.88-0.99)	0.023	1.15 (1.11-1.20)	<0.001
	60-69	2.35 (2.26-2.45)	<0.001	2.77 (2.50-3.07)	<0.001	1.36 (1.22-1.53)	<0.001	0.85 (0.80-0.91)	<0.001	0.58 (0.56-0.61)	<0.001	1.25 (1.16-1.35)	<0.001	1.70 (1.60-1.81)	<0.001
	70-79	3.43 (3.27-3.61)	<0.001	3.46 (3.07-3.89)	<0.001	1.76 (1.52-2.05)	<0.001	0.64 (0.59-0.70)	<0.001	0.36 (0.33-0.38)	<0.001	2.24 (2.00-2.51)	<0.001	1.25 (1.16-1.34)	<0.001
	>80	3.13 (2.96-3.32)	<0.001	1.76 (1.69-1.99)	<0.001	0.98 (0.81-1.19)	0.835	0.59 (0.53-0.66)	<0.001	0.52 (0.48-0.56)	<0.001	1.22 (1.09-1.38)	0.001	1.07 (1.16-1.34)	<0.001
Education level	No complete education	Referent	-	Referent	-	Referent	-	Referent	-	Referent		Referent		Referent	-
	Any education	1.17 (1.14-1.21)	<0.001	1.83 (1.69-1.99)	<0.001	1.08 (0.98-1.18)	0.111	1.26 (1.20-1.33)	<0.001	0.86 (0.83-0.89)	<0.001	0.91 (0.86-0.96)	0.001	1.07 (1.04-1.11)	<0.001
Marital status	Single/divorced/widow	Referent	-	Referent	-	Referent	-	Referent	-	Referent	-	Referent	-	Referent	-
	Married/Cohabiting	0.8 (0.78-0.83)	<0.001	1.01 (0.93-1.11)	0.785	0.62 (0.56-0.68)	<0.001	1.26 (1.20-1.33)	<0.001	0.8 (0.77-0.84)	<0.001	0.81 (0.76-0.86)	<0.001	0.84 (0.81-0.88)	<0.001

For peer review only

Wealth quintile	1	Referent	-	Referent	-	Referent	-	Referent	-	Referent	-	Referent	-	Referent	-
	2	0.83 (0.79-0.86)	<0.001	1.31 (1.14-1.52)	<0.001	1.84 (1.47-2.29)	<0.001	2 (1.83-2.20)	<0.001	0.96 (0.92-1.01)	0.105	1.06 (0.98-1.14)	0.142	1.1 (1.05-1.15)	<0.001
	3	0.99 (0.95-1.03)	0.698	1.2 (1.04-1.39)	0.014	2.36 (1.90-2.29)	<0.001	2.08 (1.90-2.78)	<0.001	0.71 (0.68-0.75)	<0.001	0.87 (0.81-0.94)	0.001	0.91 (0.87-0.95)	0.001
	4	1.3 (1.25-1.36)	<0.001	1.64 (1.42-1.88)	<0.001	6.19 (5.07-7.56)	<0.001	3.26 (2.98-3.55)	<0.001	0.51 (0.49-0.54)	<0.001	1.41 (1.30-1.53)	<0.001	1.32 (1.25-1.38)	<0.001
	5	1.6 (1.52-1.69)	<0.001	2.7 (2.34-3.12)	<0.001	11.16 (9.05-13.76)	<0.001	4.5 (4.12-4.93)	<0.001	0.39 (0.37-0.42)	<0.001	2.46 (2.23-2.73)	<0.001	1.62 (1.53-1.72)	<0.001

For peer review only

For peer review only - http://bmjopen.bmj.com/site/about/guidelines.xhtml

Appendix table 2 Demographic characteristics of participants with and without measured cholesterol (n = 2071)

parameter	group	Not measured cholesterol	Measured Cholesterol
Place of living	Rural	61.30%	61.50%
	Urban	38.70%	38.50%
Gender	Male	47.30%	40.60%
	Female	52.70%	59.4%*
Age median (IQR)		56 (46-67)	55 (47-68)
Education level	No completed education	70.10%	67.50%
	Any education	29.90%	32.30%
Marital status	Married/Cohabiting	69.20%	67.90%
	Single/widowed/divorced	30.80%	32.10%
Wealth quintile	1	21.10%	18.20%
	2	20.10%	19.80%
	3	20.80%	19.00%
	4	17.90%	23.10%
	5	20.10%	19.80%

*p<0.005

For peer review only

Appendix table 3 Univariable associations between demographic characteristics and cardiovascular risk factors (n=2071)

parameter	group	Hypertension	Diabetes	Hypercholesterolaemia	Obesity	Smoking	One CVD risk factor or incl. Cholesterol	One CVD risk factors or more exc. Cholesterol
Place of living	Rural	46.00%	2.30%	5.00%	20.40%	28.40%	75.50%	72.60%
	Urban	55.8%*	5.5%*	9.6%*	37.2%*	21.0%*	79.6%*	77.8%*
Gender	Female	54.80%	4.00%	7.90%	36.70%	8.40%	74.80%	72.30%
	Male	44.7%*	3.0%*	5.3%*	17.0%*	42.2%*	79.5%*	76.7%*
Age		0.239*	0.081*	0.080*	-0.039*	-0.114*	0.068*	0.073*
Age groups	40-49	37.50%	1.80%	4.90%	28.00%	30.60%	75.00%	71.60%
	50-59	52.20%	4.20%	7.30%	27.20%	27.60%	75.40%	75.70%
	60-69	60.50%	5.70%	9.80%	28.20%	19.10%	80.60%	76.30%
	70-79	68.00%	5.70%	9.90%	21.70%	14.70%	84.40%	80.40%
	>80	68.0%**	3.4%**	5.8%**	16.0%**	17.6%**	80.6%**	77.2%**
Education level	No completed education	48.50%	2.60%	5.60%	22.80%	24.60%	76.00%	72.90%
	Any education	52.0%*	5.4%*	8.9%*	34.3%*	27.8%*	79.1%*	77.7%*
Marital status	Married/Cohabiting	45.50%	3.20%	5.70%	25.50%	29.20%	76.30%	73.70%
	Single/divorced/ Widowed	60.6%*	4.3%*	9.4%*	29.4%*	16.1%*	79.1%*	76.7%*
Wealth quintile	1	45.80%	1.70%	1.60%	13.80%	32.00%	73.40%	71.10%
	2	41.80%	2.30%	3.10%	17.50%	32.10%	75.70%	73.40%
	3	46.90%	2.40%	3.80%	21.50%	26.60%	71.50%	70.20%
	4	54.00%	3.70%	9.50%	34.60%	20.40%	79.10%	77.50%
	5	62.4%**	7.7%**	15.0%**	50.6%**	16.0%**	86.3%**	82.2%**

*P<0.001, **P for trend<0.001

For peer review only

STROBE Statement—Checklist of items that should be included in reports of ***cross-sectional studies***

	Item No	Recommendation
**Title and abstract**	1	(*a*) Indicate the study's design with a commonly used term in the title or the abstract
		(*b*) Provide in the abstract an informative and balanced summary of what was done and what was found
**Introduction**		
Background/rationale	2	Explain the scientific background and rationale for the investigation being reported
Objectives	3	State specific objectives, including any prespecified hypotheses
**Methods**		
Study design	4	Present key elements of study design early in the paper
Setting	5	Describe the setting, locations, and relevant dates, including periods of recruitment, exposure, follow-up, and data collection
Participants	6	(*a*) Give the eligibility criteria, and the sources and methods of selection of participants
Variables	7	Clearly define all outcomes, exposures, predictors, potential confounders, and effect modifiers. Give diagnostic criteria, if applicable
Data sources/ measurement	8*	For each variable of interest, give sources of data and details of methods of assessment (measurement). Describe comparability of assessment methods if there is more than one group
Bias	9	Describe any efforts to address potential sources of bias
Study size	10	Explain how the study size was arrived at
Quantitative variables	11	Explain how quantitative variables were handled in the analyses. If applicable, describe which groupings were chosen and why
Statistical methods	12	(*a*) Describe all statistical methods, including those used to control for confounding
		(*b*) Describe any methods used to examine subgroups and interactions
		(*c*) Explain how missing data were addressed
		(*d*) If applicable, describe analytical methods taking account of sampling strategy
		(*e*) Describe any sensitivity analyses
**Results**		
Participants	13*	(a) Report numbers of individuals at each stage of study—eg numbers potentially eligible, examined for eligibility, confirmed eligible, included in the study, completing follow-up, and analysed
		(b) Give reasons for non-participation at each stage
		(c) Consider use of a flow diagram
Descriptive data	14*	(a) Give characteristics of study participants (eg demographic, clinical, social) and information on exposures and potential confounders
		(b) Indicate number of participants with missing data for each variable of interest
Outcome data	15*	Report numbers of outcome events or summary measures
Main results	16	(*a*) Give unadjusted estimates and, if applicable, confounder-adjusted estimates and their precision (eg, 95% confidence interval). Make clear which confounders were adjusted for and why they were included
		(*b*) Report category boundaries when continuous variables were categorized
		(*c*) If relevant, consider translating estimates of relative risk into absolute risk for a meaningful time period
Other analyses	17	Report other analyses done—eg analyses of subgroups and interactions, and sensitivity analyses

For peer review only

For peer review only - http://bmjopen.bmj.com/site/about/guidelines.xhtml

**Discussion**		
Key results	18	Summarise key results with reference to study objectives
Limitations	19	Discuss limitations of the study, taking into account sources of potential bias or imprecision. Discuss both direction and magnitude of any potential bias
Interpretation	20	Give a cautious overall interpretation of results considering objectives, limitations, multiplicity of analyses, results from similar studies, and other relevant evidence
Generalisability	21	Discuss the generalisability (external validity) of the study results
**Other information**		
Funding	22	Give the source of funding and the role of the funders for the present study and, if applicable, for the original study on which the present article is based

*Give information separately for exposed and unexposed groups.

Note: An Explanation and Elaboration article discusses each checklist item and gives methodological background and published examples of transparent reporting. The STROBE checklist is best used in conjunction with this article (freely available on the Web sites of PLoS Medicine at http://www.plosmedicine.org/, Annals of Internal Medicine at http://www.annals.org/, and Epidemiology at http://www.epidem.com/). Information on the STROBE Initiative is available at www.strobe-statement.org.

For peer review only
